# Adherence to antiretroviral therapy and drug resistance impact evolving viral suppression among a cohort of children and adolescents living with perinatal HIV infection in western Kenya

**DOI:** 10.1371/journal.pone.0355492

**Published:** 2026-09-15

**Authors:** Rachel Vreeman, Winstone Nyandiko, Allison DeLong, Ashley Chory, Michael Scanlon, Josephine Aluoch, Edwin Sang, Vladimir Novitsky, Festus Sang, Celestine Ashimosi, Samuel Ayaya, Eslyne Jepkemboi, Millicent Orido, Joseph Hogan, Rami Kantor

**Affiliations:** 1 Icahn School of Medicine at Mount Sinai, New York, New York, United States of America; 2 Arnhold Institute for Global Health, New York, New York, United States of America; 3 Academic Model Providing Access to Healthcare (AMPATH), Eldoret, Kenya; 4 Moi University, Eldoret, Kenya; 5 Brown University School of Public Health, Providence, Rhode Island, United States of America; 6 Global Health Program, College of Liberal Arts and Sciences, DePauw University, Greencastle, Indiana, United States of America; 7 Brown University Alpert Medical School, Providence, Rhode Island, United States of America; Chantal Biya International Reference Centre for HIV/AIDS Research on Prevention and Treatment: CIRCB, CAMEROON

## Abstract

**Background:**

We characterize antiretroviral therapy (ART) adherence among children and adolescents living with perinatal HIV (CALWH) and investigate its impact on treatment failure (TF) and drug resistance (DR).

**Methods:**

We enrolled Kenyan CALWH ≤15 years on NNRTI-based ART and monitored adherence over six months, with monthly, validated questionnaires and continuous MEMs (Medication Event Monitoring Systems) electronic dose monitoring. We assessed associations between TF (viral load (VL) >1,000 copies/mL; local guidelines) at 1 month (Blood Draw 1, BD1) and, for a subset, at 4 months (Blood Draw 2, BD2) and adherence using weighted logistic regression. Sanger genotyping identified reverse transcriptase DR mutations upon TF. Associations between number of mutations and adherence was modelled by weighted Poisson regression.

**Results:**

Among 692 CALWH (51% female; mean 8.4 years) on ART for mean 2.6 years between 2010−2013, 44% reported non-adherence at baseline. At BD1, 21% (N = 143/464) had TF. Reported non-adherence at enrollment and lower MEMS adherence between enrollment and BD1 were significantly associated with TF at BD1 (respectively, OR=1.25 per 1 unit higher non-adherence, 95% CI = 1.05 to 1.48, and OR=0.69 per 1 unit z-score higher MEMS adherence, 95% CI = 0.50 to 0.95). Among those with TF at BD1, 43% (31/72) had TF at BD2, higher MEMS adherence no longer predicted VL suppression. DR genotyping among those with TF at BD1 found fewer mutations for those without treatment interruption >48 hours (RR = 0.17, CI = 0.05 to 0.61), but among them, more mutations for those with longer interruptions (hours, log-10 RR = 2.48, CI = 1.10 to 5.59). More resistance was also associated with longer ART years (Log-10 RR = 1.47, CI = 1.15 to 1.89), and higher log-10 VL at BD1 (RR = 1.21, CI = 1.06 to 1.37)). MEMS adherence had significant, U-shaped relationship, with most mutations at lower (<50%) adherence.

**Conclusion:**

Well characterized non-adherence among Kenyan CALWH was common and resulted in increased risks for TF and extensive DR, highlighting treatment challenges.

## Introduction

The Joint United Nations Programme on HIV/AIDS (UNAIDS) estimates that 1.7 million children are living with HIV worldwide [1]. Nearly 90% of these children live within African countries, with the highest incidence in South Africa, Mozambique, United Republic of Tanzania, and Kenya [2]. In Kenya, there are between 120,000 and 230,000 children (ages 0–19) living with HIV, with 61% estimated to be on antiretroviral therapy (ART) [2,3].

Most children perinatally infected with HIV in African countries lacked access to ART until after 2000, when [USAID-PEPFAR]-supported programs expanded ART availability for HIV prevention and treatment [4]. As these programs grew between 2003–2015, many children and adolescents living with HIV (CALWH) gained access to non-nucleoside reverse transcriptase inhibitor (NNRTI)-based regimens, similar to the Kenyan cohort studies here. Despite major improvements in access and corresponding declines in pediatric HIV morbidity and mortality, many resource-limited settings (RLS) lacked capacity for detailed adherence measurement or viral load (VL) monitoring. Consequently, data linking childhood adherence to later treatment failure and drug resistance (DR) remain limited [5–7]. Treatment failure (TF), defined by viral non-suppression on ART, drives accumulation of drug resistance mutations (DRMs), reducing future treatment options and increasing morbidity and mortality [8,9]. These past challenges may also shape outcomes I n the current ART era, even on new regimens [10–16]. This study aims to characterize adherence behaviors in this first generation of CALWH “survivors”, and to fill key gaps in understanding their treatment failure and drug resistance histories as they transition into adulthood.

While it is well-established that ART adherence impacts the clinical status of children living with HIV, [17,18] achieving and sustaining robust adherence in this patient population remains complicated by a number of factors, including HIV-related stigma, [19–23] mental health challenges, [24] food insecurity, evolving child development or autonomy, socio-structural components (e.g., transportation, poverty, work/child care responsibilities), and access to consistent medical care. Importantly, nuanced adherence behaviors, such as frequency and duration of treatment interruptions or patterns of missed and late doses, have been poorly characterized [25]. This limited characterization may explain why adherence measures among CALWH do not consistently correlate with viral suppression or treatment failure [26,27]. Few studies have investigated the relationship between adherence and treatment failure in perinatally infected children in RLS, [8,28–31] and existing studies often fail to account for treatment interruptions or late dosing, which are particularly relevant in this population. [32–35].

More intensive adherence assessment strategies, such as Medication Event Monitoring Systems (MEMS®; Aardex Group), which electronically record bottle-opening dates and times, provide longitudinal data on adherence and dose timing. Measurement of plasma drug levels offers complementary insight into recent drug exposure and therapeutic adequacy, capturing the impact of pharmacokinetics (PKs) on treatment therapeutic outcome [36,37]. Integrating MEMS data, plasma drug levels, and self- or caregiver-reported adherence enables a more comprehensive assessment of how adherence behaviors, clinical status, and pharmacokinetics (PKs) may influence treatment failure and drug resistance [38].

For CALWH, we need to accurately characterize more nuanced aspects of treatment adherence, such as frequency and length of treatment interruptions or the precise number of missed doses over a given period, to understand how to prevent the significant risks of treatment failure. More intensive adherence measuring strategies, such as using MEMS, in which the date and time of each opening of the bottle caps is recorded electronically, provides additional information about longitudinal treatment adherence and dose timing. Examining plasma drug levels can characterize patients’ immediate exposure to their ART regimen and whether the level is therapeutic, allowing for assessment of the impact of the PK properties of a medication on the therapeutic outcome [36,37]. Overlaying MEMS data and plasma drug level information, in addition to self-report or caregiver-reports of adherence, offers the opportunity to detail how ART adherence, clinical status, and PKs may influence treatment failure and drug resistance [38].

We also have an ongoing data gap in terms of HIV drug resistance patterns among CALWH. Even as new regimens that may be less susceptible to non-adherence, such as integrase inhibitor-based therapies, become more available in these settings, the need to support adherence to protect limited regimen options remains critical. Understanding the patterns of drug resistance that have or have not evolved among CALWH on these previously common first-line regimens could be critical for informing subsequent ART choices, particularly as newer agents and formulations from existing and older drug classes are increasingly introduced. Thus, increasing data resolution for this patient population appears crucial, given recent evidence suggesting that small changes in adherence may significantly impact patient clinical status [39,40].

Both achieving and maintaining viral suppression while limiting drug resistance among CALWH requires a better understanding of the relationship among ART adherence, treatment failure, and drug resistance. Here, we provide in-depth analysis of these relationships in a cohort of perinatally-infected, Kenyan CALWH, using detailed adherence measurement strategies, including MEMS tracking, questionnaires, plasma drug levels and clinical biomarkers. Our objectives were to comprehensively characterize ART adherence among CALWH receiving routine clinical care and to demonstrate its impact on treatment failure and HIV drug resistance.

## Methods

### Study setting

This study was conducted within four clinics of the large Academic Model Providing Access to Healthcare (AMPATH)-Kenya care program, a collaboration between Moi University School of Medicine and Moi Teaching and Referral Hospital in Eldoret, Kenya, and a consortium of North American academic medical centers.

CALWH were followed with monthly clinical visits, at which a monthly supply of medications was distributed at no cost to patients through USAID/PEPFAR funding. The pediatric first-line ART available at AMPATH included abacavir (ABC)/zidovudine (AZT)/stavudine (D4T) plus lamivudine (3TC) plus nevirapine (NVP)/efavirenz (EFV.) At least one nurse or counselor within each of the study clinics was trained to provide pediatric ART adherence counseling.

### Study design

Between 2010 and 2013, a cohort of 706 pediatric participants and their parents or caregivers were enrolled in the phased Comprehensive Adherence Measurement for Pediatrics (CAMP) study [41–43]. Inclusion criteria for study enrollment were: 1) 0–14 years old; 2) HIV positive after vertical/perinatal transmission; 3) currently on or starting an efavirenz- (EFV) or nevirapine- (NVP) based ART regimen; and 4) engaged in care at one of four AMPATH clinics: Moi Teaching and Referral Hospital, Kitale, Webuye, or Turbo.

At baseline, participants received routine AMPATH clinical examinations (age-appropriate, standardized history taken, vital signs and physical examination), then had study evaluations that included collecting more detailed sociodemographic data, clinical histories and completing adherence measurement questionnaires. They were also initiated on continuous electronic dose monitoring of their NNRTI medication using MEMS® caps, with data downloaded at any clinical visit.. Throughout the follow-up period, we also extracted sociodemographic, clinical exam, ARV regimen and appointment-related data from the AMPATH electronic medical record system. At one or two timepoints during the six months’ follow-up, blood samples were also drawn from participants for CD4 count, plasma drug concentration, HIV viral load and, if viral load was detectable, HIV drug resistance genotyping. Whenever possible, these were timed to align with blood draws required for clinical care.

CAMP enrollment took place in two planned phases, with the only difference being in the number of blood draws done for participants. Participants in the first phase (N = 328) had two blood draws during the course of follow-up, providing the first blood sample 1–3 after enrollment (average length of time = 2.0 months) and then a second blood draw 1–3 months after the first (average 2.9 months after first blood draw.) The second phase of enrolled participants (N = 368), which was funded through a different source, only had one blood draw done, at 1–3 months after enrollment. All study blood samples were processed for CD4 percentage/count evaluation and then any remaining plasma was frozen and stored in the AMPATH biorepository for future plasma drug concentrations, viral load measurement and resistance genotyping to be done -- the results presented here. These evaluations were not available in the AMPATH clinical system during 2010–2013 and the original CAMP study (1K23MH087225, PI: Vreeman) did not have funding to complete these additional lab investigations, only to store the samples. The evaluations were done after obtaining additional funding (R01AI120792, MPI: Vreeman/Kantor) for this purpose in 2016, when sample evaluations began.

For this analysis, we make use of data from up to 3 study visits per participant, corresponding to (1) CAMP enrollment, where the adherence questionnaire was first delivered and MEMS® monitoring initiated, (2) first blood draw (BD 1), which also included the adherence questionnaire, download of MEMS® data and a blood draw for drug levels and virologic outcomes, and (3) among participants in the first CAMP phase, the second blood draw visit (BD 2), where there was again administration of the CAMP adherence questionnaire and MEMS® download.

### Ethical approvals

The study protocol was approved by the Institutional Review Boards of Indiana University School of Medicine in Indianapolis, Indiana, and the Miriam Hospital in Providence, Rhode Island, and by the Institutional Scientific and Ethical Review Committee at Moi University School of Medicine and Moi Teaching and Referral Hospital in Eldoret Kenya.

### Independent variables (Adherence measures)

Three adherence measurements were used to quantify participant adherence to give comprehensive data about both medication-taking behaviors and whether the exposure to ART was therapeutic or sub-therapeutic: 1) validated caregiver or self-report questionnaires [43–46] (CAMP questionnaire, included in Supplemental Table A), 2) electronic dose monitoring, and 3) plasma drug levels. These multiple measures offered both short-term and longer-term adherence data, while also accounting for social determinants related to adherence behaviors. The CAMP questionnaire, a short, 10-item adherence questionnaire, constructed and validated in this setting and with this population, was administered at enrollment and each subsequent study visit [42,47]. Caregiver questionnaires were administered to the caregiver who had primary responsibility for overseeing the child’s medication-taking in the past month. For children with any medication-taking responsibility, child questionnaires were also administered. The written questionnaire was administered verbally, in Kiswahili or English, by an experienced research assistant at each study visit.

Electronic dose monitoring was conducted using MEMS® bottle caps on children’s NVP or EVP, both tablet and liquid formulations, providing additional, specific data on dose timing, patterns of adherence, and treatment interruptions. Patients were informed of the purpose of MEMS® and instructed in care of the cap and bottle. MEMS adherence data were recorded continuously throughout study follow-up, with downloading and viewing of the data throughout follow-up, including at Blood Draw 1 (BD1) and Blood Draw 2 (BD2) to calculate measurements for the period between enrollment and BD1 and the period between BD1 and BD2. Dosing time data recorded by the MEMS® were read into PowerView software (Version 3.5.2; AARDEX, Inc.) and then converted into a SAS dataset for further analysis (Version 9.4; SAS Institute, Inc., Cary North Carolina).

Finally, we measured plasma drug concentration for NVP or EFV, corresponding to the child’s regimen. Plasma drug levels were analyzed and classified as therapeutic (NVP 3.0–7.6 µg/mL or EFV 1.0–4.0 µg/mL), sub-therapeutic (NVP < 3.0 µg/mL or EFV < 1.0 µg/mL), or supra-therapeutic (NVP > 7.6 µg/mL or EFV > 4.0 µg/mL), based on prior PK studies in similar patient populations.^42–45^

These measurements were used to generate 7 adherence measures used as independent variables in the analyses: (1) the value of the adherence questionnaire score at the time of the blood draw (range 0–6, higher meaning worse adherence), (2) the value of adherence questionnaire score from the prior visit (i.e., enrollment or BD1), (3) percent adherence using MEMS in the period between visits and immediately before the BD, (4) the number of treatment interruptions >48 hours in the period between visits leading up to the BD standardized by the number of weeks in the period, measured by MEMS®, (5) duration in hours of the maximum treatment interruption between visits leading up to the BD, measured by MEMS®, (6) a dichotomous variable for whether there was at least 1 interruption >48 hours (to stabilize the correlated continuous measures 4 and 5), and (7) plasma EFV or NVP drug level category at the time of the blood draw. Two measures were transformed so as to have more stable standard errors: percent adherence was transformed by adding 1, dividing by 102 and converting to a z-score, and the duration of the maximum treatment interruption was log10 transformed. As an illustration, the z-score for 98% MEMS adherence is approximately 2, 2% is −2, and 50% is 0 (Appendix A).

### Laboratory methods

VL testing was done at the AMPATH Reference Lab in Eldoret, Kenya between 2016−2018 using Abbott’s RealTime assay with the Molecular m2000sp for sample preparation and m2000rt for real-time amplification/detection (Abbott Molecular Inc., Des Plaines, IL). Based on World Health Organization definitions, detectable VL was defined to be > 40 copies/mL, and TF was defined to be > 1000 copies/mL [48]. Plasma NVP and EFV concentrations were measured using a rapid, automated enzyme immunoassay (EIA) developed by ARK Diagnostics in Sunnyvale, California, USA [49]. The ARK NVP assay is based on competitive binding to antibody between drug in the sample and drug-labeled enzymes. Drug concentration is measured spectrophotometrically (Hitachi 902® bench top analyzer) in terms of enzyme activity. The EIA shows good correlation with high performance liquid chromatography (HPLC) and provides a cost-effective way to determine NVP and EFV concentrations in areas with high HIV prevalence and limited testing resources [50,51]. Sanger sequencing of partial HIV-1 pol to evaluate drug resistance mutations was performed on all plasma specimens with detectable viral load (>40 copies/mL). NRTI and NNRTI mutations were identified by Stanford tools. More detailed descriptions of the drug resistance genotyping for these samples is described in a prior publication [52].

All study samples were consistently stored at −80 °C in Kenya and the United States until testing, with minimal freeze-thaw cycles and continuous freezer monitoring and quality assurance. Samples were shipped between continents exclusively on dry ice and handled according to best laboratory practices at all sites. Prior to testing, samples were thawed once and tested immediately to minimize degradation, ensuring validity for viral load, drug resistance, and drug-level analyses.

### Outcomes

The three main outcomes were TF at BD1, TF at BD2, and the number of NRTI and NNRTI resistance mutations at BD1 among those with TF and a genotype.

### Statistical methods

We used generalized additive and linear weighted models to quantify the effect of medication adherence measures on each outcome. TF (yes/no) was modeled using logistic regression and number of resistance mutations was modeled using a Poisson regression with overdispersion. All models were adjusted for gender, age, time on ARV treatment, and orphan status. The models of TF at BD2 were additionally adjusted for VL at BD1, log10 transformed. Because treatment failure at BD2 was highly associated with TF at BD1, and because there were only 9 participants with treatment failure at BD2 among the 136 suppressed at BD1, we examined TF at BD2 just among those failing at BD1. We used inverse probability weighting in fitting the models to address missing data due to incomplete follow up or VL test results at BD1 and BD2 (details below). Summary statistics for individual variables are presented as mean (standard deviation (SD)) for numeric measures and proportions for categorical or ordinal measures.

### Inverse probability weights for handling incomplete data

Inverse probability weighting was used to account for potential bias due to missing VL results and incomplete follow up at BD1 and BD2. For failure and resistance outcomes at BD1, the weight was the inverse of the probability of having a VL measure conditional on baseline demographic covariates. For failure at BD2, the weights were computed sequentially over visits using the product of (i) probability of having a measured VL at BD1 (as described above), (ii) probability of having a BD2 visit conditional on having a VL measurement at BD1, and [3] probability of having a VL measurement at BD2 conditional on having a blood draw at visit 2 and having a measured viral load at visit 1. These probabilities were estimated using generalized additive models; each model included baseline covariates and prior values of viral load as predictor variables. Our approach corresponds to assuming sequential missing at random, or that the probability of missing a VL measurement can be explained by baseline covariates and prior values of VL.

### Regression model specifications

All models were first fit using penalized generalized additive models (GAM) [53] using the ‘mgcv’ package [54] in R version 4.0.2 [55]. GAM allows the effect of each covariate to be nonlinear. When the estimated relationship was linear, the odds ratio (OR) or rate ratio (RR) and 95% CI are shown, and when the relationship was non-linear, a curve showing the effect of the covariate and 95% CI on the log-odds scale is provided. We used transformed versions of the covariates to ensure model stability. For BD2, we collapsed maternal and paternal orphans into a “single orphan” category due to small sample size and omitted the duration of the longest treatment interruption variable due to instability and collinearity with the number of treatment interruptions variable (correlation = 0.75).

## Results

**Fig 1:**
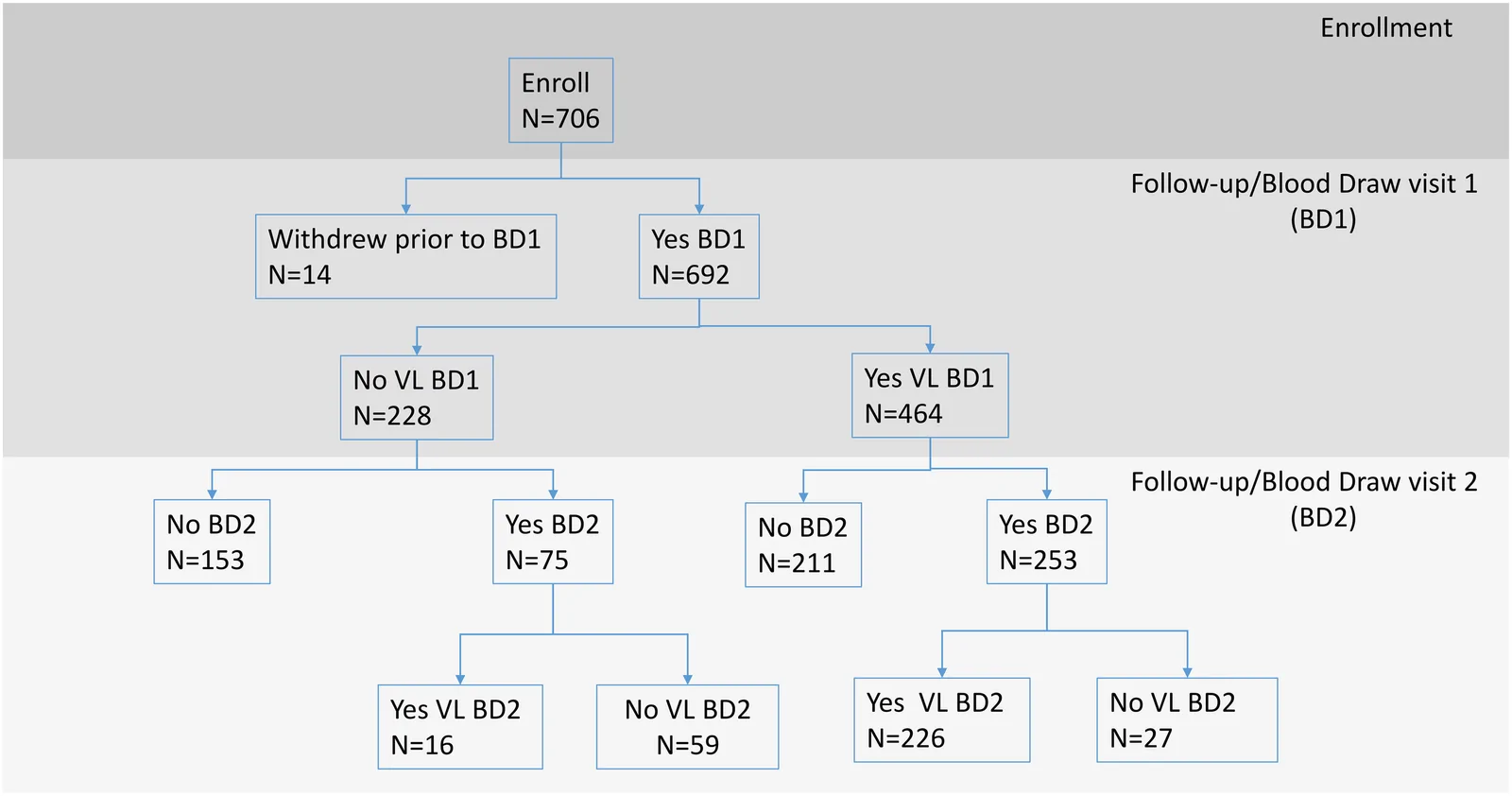
Flow of Participants in Study. This figure shows the (A) amount of participants and (B) viral load status at every stage of the study.

Of the 706 perinatally HIV-infected children enrolled, 14 withdrew from the study and are omitted from analyses, and 692 had at least one blood draw (Fig 1) that was eligible for inclusion in these analyses. At CAMP enrollment, the average age of the 692 study participants was 8.4 years, 51% were female, and they had been on ART for a mean of 2.6 years (Table 1). All participants were on nevirapine (NVP)- or efavirenz (EFV)-based ART regimens, the two most common of which combined an NNRTI with abacavir and lamivudine. At enrollment, 13% were double orphans with both parents dead, and 21% had lost one biological parent. Boys and girls had similar covariate profiles.

**Table 1:** Demographic characteristics of study participants at enrollment.

Variable	Total (N = 692)	Male (N = 336)	Female (N = 356)
**Age at enrollment, years, mean (SD)**	8.4 (3.3)	8.4 (3.3)	8.4 (3.3)
**Age group at enrollment, years**			
<5	126 (18)	59 (18)	67 (19)
≥5 to <10	341 (49)	169 (50)	172 (48)
≥10	225 (33)	108 (32)	117 (33)
**Orphan status at enrollment**			
Double orphan	87 (13)	40 (12)	47 (13)
Maternal orphan	75 (11)	35 (10)	40 (11)
Paternal orphan	72 (10)	38 (11)	34 (10)
Non-orphan	457 (66)	223 (66)	234 (66)
**Years on ART at enrollment**	2.6 (2.0)	2.7 (1.9)	2.6 (2.0)
**ART regimen**			
3TC/ABC/EFV or NVP	407 (59)	191 (57)	216 (61)
3TC/D4T/EFV or NVP	108 (16)	58 (17)	50 (14)
AZT/3TC/EFV or NVP	167 (24)	83 (25)	84 (24)
Other	8 (1)	4 (1)	4 (1)

**Notes**: Unless otherwise noted, all results are presented as N (%). Percentages are based on non-missing data. Missing data: orphan status (n = 1), years on ART (n = 2), ART regimen (n = 2). Other ART regimens: 3TC/EFV or NVP/TDF (n = 6); AZT/ABC/EFV or NVP (n = 1); D4T/ABC/EFV or NVP (n = 1).

**Abbreviations:** ART, antiretroviral therapy; ABC, abacavir; AZT, zidovudine; D4T, stavudine; EFV, efavirenz; NVP, nevirapine; TDF, tenofovir; 3TC, lamivudine; SD, standard deviation.

At enrollment, only 10% knew their own HIV status and 44% of participants reported non-adherence on at least one measurement item on the CAMP questionnaire (Table 2). Responses revealed various degrees and types of non-adherence: 11% reported having problems taking their ART, 20% reported problems “keeping time” when taking their ART, 10% reported missing 1 or more ART doses in the past week, and 10% reported missing 1 or more ART doses in the last month.

**Table 2:** Reported Adherence on CAMP Questionnaires at Enrollment.

Variable	Total (N = 692)	Male (N = 336)	Female (N = 356)
**Adherence questionnaire completed by**			
Caregiver	541 (78)	252 (75)	289 (81)
Child/adolescent	20 (4)	9 (3)	11 (3)
Missing	131 (19)	75 (22)	56 (16)
**Relationship of questionnaire respondent**			
Mother	323 (47)	144 (43)	179 (50)
Father	53 (8)	29 (9)	24 (7)
Grandparent	50 (7)	27 (8)	23 (6)
Other relative	67 (10)	31 (9)	36 (10)
Non-related caregiver	17 (2)	8 (2)	9 (3)
Self	20 (3)	9 (3)	11 (3)
Other	28 (4)	11 (3)	17 (5)
Missing	134 (19)	77 (24)	57 (16)
**Aware of own HIV status at enrollment (Q2)**			
Yes	69 (10)	34 (10)	35 (10)
No	478 (69)	217 (65)	261 (73)
Missing	145 (21)	85 (25)	60 (17)
**Problems “keeping time” when taking medicines (Q4)**			
Yes	139 (20)	68 (20)	71 (20)
No	418 (60)	191 (57)	227 (64)
Missing	135 (20)	77 (23)	58 (16)
**Ever has problems taking medicines (Q5)**			
Yes	79 (11)	32 (10)	47 (13)
No	471 (68)	226 (67)	245 (69)
Missing	142 (21)	78 (23)	64 (18)
**Missed doses in past week (Q9)**			
0	484 (70)	229 (68)	255 (72)
1	34 (5)	13 (4)	21 (6)
>1	37 (5)	16 (5)	21 (6)
Missing	137 (20)	78 (23)	59 (17)
**Late doses in past week (Q10)**			
0	430 (62)	202 (60)	228 (64)
1	37 (5)	26 (8)	11 (3)
>1	82 (12)	28 (8)	54 (15)
Missing	143 (21)	80 (24)	63 (18)
**Missed doses in past month (Q12)**			
0	477 (69)	225 (67)	252 (71)
1	28 (4)	10 (3)	18 (5)
>1	40 (6)	19 (6)	21 (6)
Missing	147 (21)	82 (24)	65 (18)
**Number of non-adherent measures, mean (SD) (Q2, Q4-5, Q9-10, Q12)**	1.0 (1.2)	0.9 (1.1)	1.0 (1.3)
Missing	131	75	56
**Number of non-adherent measures (categorical)**			
0	263 (38)	121 (36)	142 (40)
1	156 (23)	75 (22)	81 (23)
>1	142 (21)	65 (19)	77 (22)
Missing	131 (19)	75 (22)	16)

**Notes**: Unless otherwise noted, all results are presented as N (%). Percentages are calculated using the total number of participants in each column. Adherence variables were derived from the CAMP Adherence Questionnaire (Supplemental Table A); question numbers shown in parentheses correspond to questionnaire items. “Missing” includes responses of “don’t know,” “refused,” or questionnaires that were not completed.

**Abbreviations:** SD, standard deviation.

Over the study period, 364 (52%) had one blood draw (BD1) and 328 (48%) had two (BD1 and BD2). Most participants with only one blood draw (358/364, 98%) were from the second study phase for which only one blood draw was planned, but the remaining 6 participants should have had a second blood draw but missed their follow-up. Those with 1 BD had longer mean time from enrollment to BD1 (2.2 vs 1.8 months) and differed by orphan status and years on ART (Supplemental Table B). Although all 692 participants had at least one blood draw during the study period, not all of these samples had enough specimen stored for the later testing, resulting in no VL test results for 228/692 (33%) of participants at BD1 and 86/328 (26%) at BD2. Those with and without VL results at BD1 were similar on most covariates; those with VL results had shorter time between enrollment and BD1 assessment (2.5 vs 1.7 months, Supplemental Table C).The regression analysis is therefore based on 464/692 with VL results from BD1 and 226/328 with VL results from BD1 and BD2 (Fig 1). Inverse probability weighting accounts for observed differences in participant characteristics for those with and without measured VL.

Using the electronic dose monitoring data (MEMS), in the period between enrollment and BD1, 43% of all participants were less than 90% adherent with dose timing, including 18% with %MEMS adherence between 75–90%, 16% with %MEMS adherence between 40–75%, and 9% with %MEMS adherence between 0- < 40%. Thirty one percent of participants also had at least one interval of 48 hours where their medication bottle was not opened. At BD1, 321 participants had viral suppression with VL≤1,000 copies/ml, and 143 were in TF (Table 3). All relationships between TF and the adherence measures appeared linear on the log-odds scale and the odds ratios (ORs) are provided in Table 4. Lower percent MEMS adherence was associated with TF (OR=0.69 per 1-unit higher MEMS adherence z-score, 95% CI = 0.50 to 0.95), as was higher non-adherence on the CAMP questionnaire at enrollment (OR=1.25 per 1-unit higher non-adherence, 95% CI = 1.05 to 1.48). Among other characteristics, we found that more years on ART (OR=2.00 per 1 log unit higher, CI = 1.17 to 3.42) and younger age (OR=0.93 per year older, CI = 0.87 to 1.00) also were associated with TF. Supplemental Table D holds summaries of the model covariates, stratified by having TF at BD1.

**Table 3:** Outcomes measured at blood draw 1 and blood 2, stratified by treatment failure status at blood draw 1.

Variable	Total (N = 464)	VL ≤ 1,000 at BD1 (N = 321)	VL > 1,000 at BD1 (N = 143)
**Genotype available at BD1**			
Yes (%)	124 (27)	4 (1)	120 (84)
No (%)	340 (73)	317 (99)	23 (16)
**Number of NRTI mutations at BD1**			
Mean (SD)	2.6 (1.7)	1.0 (1.4)	2.7 (1.7)
Missing	340	317	23
**Number of NNRTI mutations at BD1**			
Mean (SD)	2.1 (1.1)	2.2 (1.7)	2.1 (1.1)
Missing	340	317	23
**Total number of resistance mutations at BD1**			
Mean (SD)	4.7 (2.4)	3.2 (3.0)	4.8 (2.4)
Missing	340	317	23
**Treatment failure at BD2**			
Yes (%)	44 (19)	13 (8)	31 (43)
No (%)	182 (81)	141 (92)	41 (57)
Missing	238	167	71

**Notes:** Categorical measures are n (%), continuous measures are presented as mean (SD). Percentages are calculated using non-missing data within each column. Genotype and resistance mutation measures are only available for participants with a genotype at BD1.

**Abbreviations:** VL, Viral Load; BD1, blood draw 1; BD2, blood draw 2; VL, viral load; NRTI, nucleoside reverse transcriptase inhibitor; NNRTI, non-nucleoside reverse transcriptase inhibitor; SD, standard deviation.

**Table 4:** Linear components of generalized additive models for treatment failure (TF) and drug resistance mutations (DRM).

Variable	TF at BD1 N = 345		TF at BD2*** N = 56		Number of DRM at BD1 N = 91	
	OR (95% CI)	p	OR (95% CI)	p	RR (95% CI)	p
% adherence using MEMS (z-score)†	0.69 (0.50, 0.95)	0.025	0.39 (0.14, 1.10)	0.074	See Fig 3	—
Number treatment interruptions >48 hours standardized by number of weeks in period†*	1.25 (0.35, 4.52)	0.733	0.47 (0.00, 1052.5)	0.848	0.75 (0.45, 1.22)	0.246
No treatment interruption >48 hrs (MEMS)†	2.33 (0.11, 50.32)	0.589	0.72 (0.10, 5.39)	0.752	0.17 (0.05, 0.61)	0.009
Duration (hours, log10) of maximum treatment interruption†*	1.13 (0.42, 3.05)	0.808	—	—	0.63 (0.42, 0.94)	0.026
Duration (hours, log10) of maximum treatment interruption†**	0.61 (0.09, 4.11)	0.613	—	—	2.48 (1.10, 5.59)	0.031
Adherence questionnaire score (enrollment)	1.25 (1.05, 1.48)	0.013	—	—	0.98 (0.92, 1.04)	0.447
Adherence questionnaire score (BD1)	0.94 (0.76, 1.17)	0.586	1.15 (0.78, 1.68)	0.481	1.07 (0.98, 1.17)	0.117
Adherence questionnaire score (BD2)	—	—	0.53 (0.32, 0.86)	0.010	—	—
Supra-therapeutic vs sub-therapeutic drug level†	0.94 (0.48, 1.82)	0.848	0.92 (0.25, 3.40)	0.904	1.58 (1.11, 2.27)	0.014
Therapeutic vs sub-therapeutic drug level†	1.35 (0.70, 2.59)	0.367	1.12 (0.30, 4.25)	0.862	2.11 (1.45, 3.08)	<0.001
log10 years on ART	2.00 (1.17, 3.43)	0.012	0.53 (0.12, 2.31)	0.398	1.47 (1.15, 1.89)	0.003
Per year older	0.93 (0.87, 1.00)	0.044	1.00 (0.85, 1.18)	0.963	0.98 (0.95, 1.01)	0.300
Male vs female	1.00 (0.66, 1.50)	0.981	1.84 (0.67, 5.06)	0.236	1.10 (0.93, 1.30)	0.249
Double orphan vs non-orphan	1.07 (0.57, 2.01)	0.824	1.19 (0.28, 5.13)	0.816	0.95 (0.74, 1.22)	0.700
Maternal orphan vs non-orphan	0.50 (0.22, 1.13)	0.096	—	—	0.93 (0.61, 1.41)	0.720
Paternal orphan vs non-orphan	1.12 (0.60, 2.08)	0.727	—	—	0.81 (0.63, 1.05)	0.110
Single orphan vs non-orphan	—	—	2.39 (0.74, 7.75)	0.146	—	—
Log10 VL at BD1	—	—	See Fig 3	—	1.21 (1.06, 1.37)	0.005

**Notes**: Values shown are adjusted odds ratios (OR) or risk ratios (RR) with 95% confidence intervals (CI) from generalized additive models. † MEMS-derived covariates are from enrollment to BD1 for BD1 outcomes (TF at BD1 and DRM at BD1); and from BD1 to BD2 for the outcome TF at BD2.

*Among participants with at least one treatment interruption >48 hours. **Among participants without treatment interruption >48 hours.

*** TF at BD2 models restricted to participants with TF at BD1 and covariates are from BD1 to BD2

Non-linear effects are shown in Fig 3. **Abbreviations:** TF, treatment failure; DRM, drug resistance mutations; BD1, blood draw 1; BD2, blood draw 2; VL, viral load; ART, antiretroviral therapy; MEMS, Medication Event Monitoring System.

**Fig 2:**
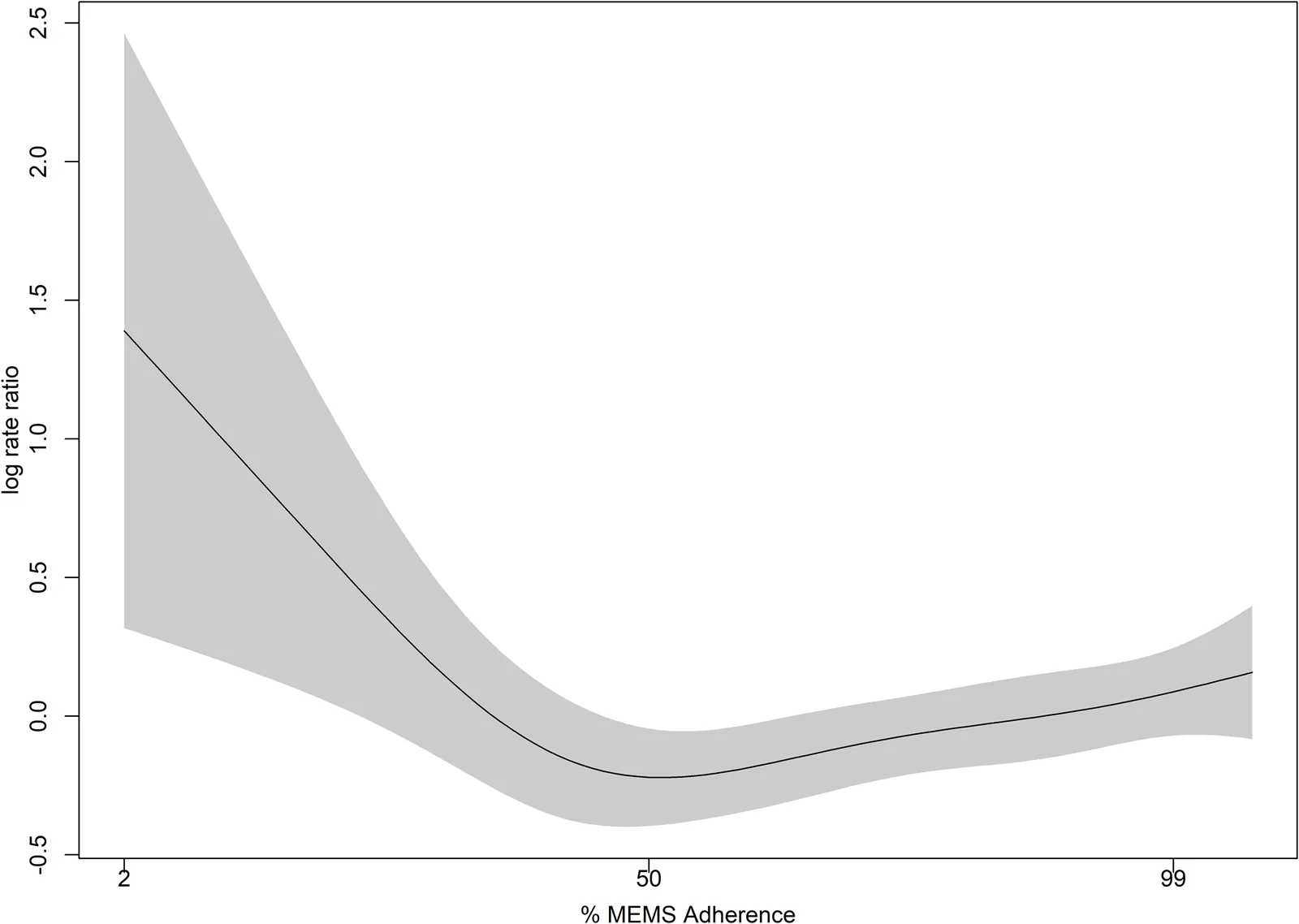
Relationship between Drug Resistance Mutations and Adherence. Shows the non-linear relationship between (a) number of drug resistance mutations (DRM) at blood draw 1 (BD1) and % Adherence by MEMS.

Drug resistance genotyping at BD1 was completed for 124 participants, including 120 of the 143 participants with TF (84%) and 4 (1%) of the 321 with VL≤1,000 copies/mL (Table 3). Participants had, on average, 2.6 NRTI DRMs, 2.1 NNRTI DRMs and 4.7 DRMs overall. Ninety-one participants with genotypes had complete covariate data and were used in the regression analysis. Percent MEMS adherence had a U-shaped relationship to the number of DRMs at BD1, with the largest number of DRMs observed at the lowest values of % MEMS adherence (Fig 2). The rate ratios (RR) for the other variables are provided in the third column of Table 4. The number of mutations at BD1 was greater for those with treatment interruptions (RR = 0.17 for those with no interruption vs 1 or more, CI = 0.05 to 0.61), shorter maximum treatment interruptions among those with an interruption >48 hours (RR = 0.63 per 1-unit log10 treatment interruption, CI = 0.42 to 0.94), those with more time on ART (OR=1.47 per 1 log10 unit, CI = 1.15 to 1.89), longer maximum treatment interruptions among those without a 48-hour interruption (RR = 2.48 per 1-unit log10 treatment interruption, CI = 1.10 to 5.59), and those with higher VL at BD1 (OR=1.21 per 1 log10 unit, CI = 1.06 to 1.37). Having therapeutic or supra-therapeutic plasma drug levels of ART was associated with more DRMs, compared sub-therapeutic ART levels (OR=2.11, CI = 1.45 to 3.08 and OR=1.58, CI = 1.11 to 2.27, respectively). Supplemental Table E holds summaries of the model covariates, stratified by the number of DRMs at BD1.

**Fig 3:**
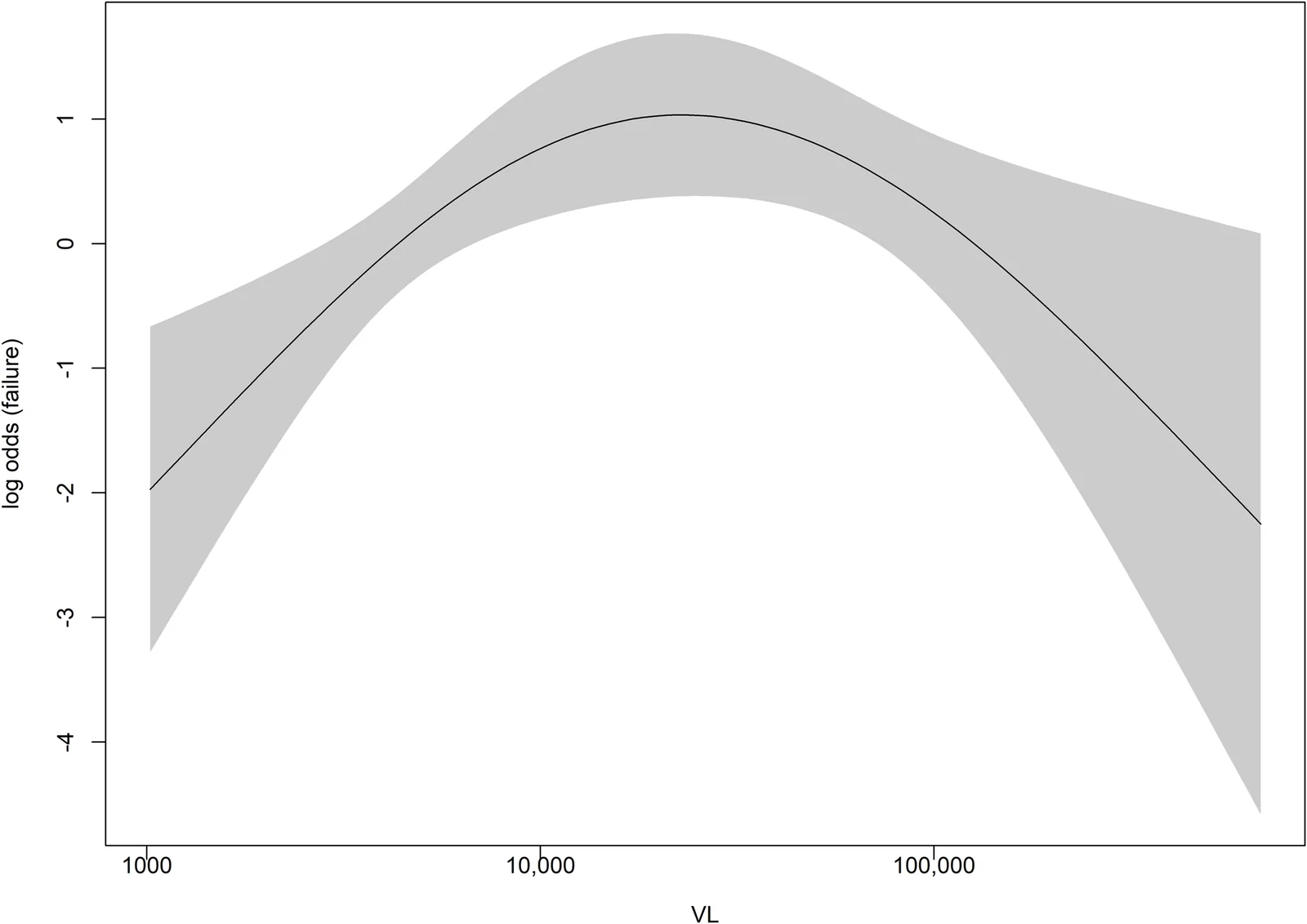
Nonlinear Relationship Between Treatment Failure at Blood Draw 2 and Viral Load at Blood Draw 1. Shows the non-linear relationship between (A) treatment failure (TF) at blood draw 2 (BD2) and (B) viral load (VL) at blood draw 1 (BD1) among those in treatment failure at blood draw 1 (BD1).

Among the 143 participants with TF at the BD1, 72 had VL information at BD2; 41 (57%) resuppressed and 31 (43%) continued to have TF (Table 3). Of these 72, 56 had complete covariate information for the regression models and 62% still had TF at BD2. Among those who continued to have TF at BD2, higher MEMS adherence no longer significantly predicted likelihood of suppressing VL to <1000 copies/mL. Among these participants, after adjusting for the questionnaire adherence score at BD1, having a higher score on the CAMP adherence questionnaire (indicating more reported non-adherence) at BD2 was associated with a lower rate of TF at BD2 (OR=0.53, CI = 0.32 to 0.86, p = 0.010) (Table 4). OR and CI for other covariates are reported in Table 4; the effect of VL at BD1 is shown in Fig 3. Supplemental Table F holds summaries of the model covariates, stratified by having TF at BD2.

## Discussion

This study shows substantial non-adherence to ART among Kenyan CALWH in routine clinical care. Importantly, non-adherence was associated with both treatment failure and HIV drug resistance, and some of its adverse effects appeared to persist over time. Globally, more than 2 million CALWH are expected to require lifelong ART, highlighting the need for effective, comprehensive, and person-centered adherence support strategies. Our findings can may help inform such strategies in similar settings, particularly where access to comprehensive adherence monitoring or viral load and drug resistance genotyping is limited.

While other evaluations, including in this Kenyan setting, [56] report higher rates of ART adherence, those estimates are often based on commonly used but unvalidated approaches that ask patients or caregivers only about missed doses and likely fail to capture true adherence behaviors. Even when electronic dose monitoring is not feasible, use of validated self-report measures that assess a broader range of contextual and behavioral factors yields greater disclosure of non-adherence than is typically reported. Given well-known biases against disclosing non-adherence, higher reported rates are generally assumed more accurate. Consistent with this, we found that poor pediatric adherence, measured using our comprehensive assessment strategy, was associated with treatment failure. Notably, adherence measures capturing more nuanced behaviors, such as a low on-time bottle opening detected by electronic monitoring, most strongly predicted TF.

Our findings illuminate critical nuances in how variations in adherence result in TF and the development of drug resistance over time. For those CALWH with multiple blood samples taken over time, many continued to have TF, even after their adherence improved. This points to the potential role of DRMs as potential mechanisms mediating treatment response in this population, an issue that is still often missed in settings where drug resistance genotyping is limited. Previous studies in adults living with HIV in RLS have reported high levels of drug resistance following TF, [57–59] which may impact the long-term efficacy of ART regimens despite adequate adherence. While this is often assumed to be the same for CALWH, there are still limited pediatric studies evaluating the impact of drug resistance on viral failure [60]. The initial findings from this cohort demonstrate a high degree of drug resistance, and our results here support the hypothesis that CALWH are similarly affected by prolonged treatment due to poor adherence [52].

Our detailed evaluations of dose timing using electronic dose monitoring also revealed complex nuances in how the duration of treatment interruptions for NNRTI-based therapy may have varying impacts on treatment failure and the development of drug resistance. We examined interactions between having at least one 48-hour interruption with both the number of 48-hour interruptions for a participant and the duration of the longest interruption. Using this strategy, we found a complex relationship, where among those with shorter interruptions (<48 hours), the longer the interruption in that window up to 48-hours, the more DR mutations the participant had. For those with longer interruptions (>48-hours), an even longer duration was protective against DR mutations. These results suggest relatively rapid resistance development in regimens anchored by antiretrovirals with low resistance barriers, such as NVP or EFV, and in regimens comprising drugs with differing half lives, where abrupt cessation can lead to functional mono- or dual-therapy and promote resistance emergence [61].The lack of DR mutations with longer ART gaps likely reflects discontinuation of all ART during that period and potential reversion of the virus to wild-type [62].

Another area for potential additional evaluation is the impact of participants’ weight or BMI; pediatric ART dosing relies on weight-based dose guidelines, and CALWH on extreme ends of the weight-groupings may have higher or lower drug exposure relative to the target levels, which may affect toxicity or efficacy, as well as resistance [63]. Continuing to examine the interplay of adherence, drug levels, and drug resistance genotypes over time are critical next steps, especially once new regimens are introduced.

These analyses have several limitations. First, the cohort consisted exclusively of children with perinatal HIV infection who initiated EFV- or NVP-based regimens. While NNRTI-based first-line regimens are increasingly being replaced, these findings remain clinically relevant. Many now-adolescents and young adults with perinatal were started on similar regimens and may have experienced comparable patterns of non-adherence, TF and drug resistance. Importantly, non-adherence and NNRTI-associated resistance may contribute to influence outcomes even after transition to integrase inhibitor-based regimens, as emerging data link prior NNRTI resistance with longer-term failure of integrase inhibitor-containing ART [64]. Furthermore, rollout of preferred agents such as dolutegravir, has been slower than desired or recommended in many settings and NNRTI-based regimens remain in use, particularly in RLS where access to newer ART options are limited. Beyond regimen type, the medication-taking patterns described here are relevant across ART regimens and may highlight adherence challenges that require support even for therapies with higher barriers to resistance. In addition, this cohort reflects children entering adolescence, a developmental period associated with increasing adherence challenges that often intensify as youth transition into young adulthood, highlighting the need proactive adherence support strategies [65,66]. Another potential limitation is that the intensity of research evaluation (compared with routine clinical care) may have increased adherence through participant awareness or more frequent study interactions. Despite this potential bias toward improve adherence, substantial non-adherence persisted and remained strongly predictive of TF. Finally, samples were stored for extended periods, precluding contemporaneous testing and potentially contributing to degradation and missing data that could affect interpretation. Relatedly, not all stored biospecimens could undergo the full set of laboratory analyses; however, supplemental analyses did not suggest systemic differences between participants with evaluable samples and those whose samples could not be evaluated.

In conclusion, comprehensively assessed poor adherence among Kenyan children and adolescents living with HIV was associated with longitudinal treatment failure and drug resistance. To maintain our gains in keeping children and adolescents with perinatal HIV infection alive and to prolong the impact of newer ART regimens, we must monitor adherence comprehensively and continue to invest in interventions to support adherence. The extent of treatment failure and drug resistance on traditional NNRTI-based regimens compels us to continue to push for increased access to drug resistance genotyping and new ART regimens if we are to sustain long-term ART success in this vulnerable population and truly end pediatric AIDS.

## Supporting information

S1 Table CAMP Adherence Questionnaire. (DOCX)

S2 Table Characteristics of participants at CAMP enrollment and Blood Draw 1, stratified by number of blood draws completed. (DOCX)

S3 Table Comparison of participants with and without viral load (VL) results at blood draw 1 (BD1). (DOCX)

S4 Table Bivariate summaries of model covariates used in the generalized additive model for treatment failure at Blood Draw 1, stratified by viral load outcome. (DOCX)

S5 Table Bivariate summaries of model covariates stratified by number of drug resistance mutations at Blood Draw 1. (DOCX)

S6 Table Bivariate summaries of model covariates stratified by viral load suppression at Blood Draw 2. (DOCX)

S1 Appendix A: Transformation of % adherence using MEMS. (DOCX)

## References

[1] UNAIDS. Global HIV & AIDS statistics — Fact sheet. 2024.

[2] UNAIDS. Global AIDS Monitoring 2018, UNAIDS 2018 estimates and UNICEF Global Databases of nationally representative population-based surveys 2010–2017. 2018.

[3] Ng’eno B, Mwangi A, Ng’ang’a L, Kim AA, Waruru A, Mukui I, et al. Burden of HIV infection among children aged 18 months to 14 years in Kenya: results from a nationally representative population-based cross-sectional survey. J Acquir Immune Defic Syndr. 2014;66 Suppl 1(Suppl 1):S82-8. doi: 10.1097/QAI.0000000000000118 24732823 PMC4784690

[4] Chun HM, Dirlikov E, Cox MH, Sherlock MW, Obeng-Aduasare Y, Sato K, et al. Vital Signs: Progress Toward Eliminating HIV as a Global Public Health Threat Through Scale-Up of Antiretroviral Therapy and Health System Strengthening Supported by the U.S. President’s Emergency Plan for AIDS Relief - Worldwide, 2004-2022. MMWR Morb Mortal Wkly Rep. 2023;72(12):317–24. doi: 10.15585/mmwr.mm7212e1 36952290 PMC10042617

[5] Orrell C, Levison J, Ciaranello A, Bekker L-G, Kuritzkes DR, Freedberg KA, et al. Resistance in pediatric patients experiencing virologic failure with first-line and second-line antiretroviral therapy. Pediatr Infect Dis J. 2013;32(6):644–7. doi: 10.1097/INF.0b013e3182829092 23303240 PMC3881960

[6] Muri L, Gamell A, Ntamatungiro AJ, Glass TR, Luwanda LB, Battegay M, et al. Development of HIV drug resistance and therapeutic failure in children and adolescents in rural Tanzania: an emerging public health concern. AIDS. 2017;31(1):61–70. doi: 10.1097/QAD.0000000000001273 27677163 PMC5131685

[7] Barth RE, Tempelman HA, Smelt E, Wensing AMJ, Hoepelman AI, Geelen SP. Long-term outcome of children receiving antiretroviral treatment in rural South Africa: substantial virologic failure on first-line treatment. Pediatr Infect Dis J. 2011;30(1):52–6. doi: 10.1097/INF.0b013e3181ed2af3 20631647

[8] Nachega JB, Marconi VC, van Zyl GU, Gardner EM, Preiser W, Hong SY, et al. HIV treatment adherence, drug resistance, virologic failure: evolving concepts. Infect Disord Drug Targets. 2011;11(2):167–74. doi: 10.2174/187152611795589663 21406048 PMC5072419

[9] Hosseinipour MC, Gupta RK, Van Zyl G, Eron JJ, Nachega JB. Emergence of HIV drug resistance during first- and second-line antiretroviral therapy in resource-limited settings. J Infect Dis. 2013;207 Suppl 2(Suppl 2):S49-56. doi: 10.1093/infdis/jit107 23687289 PMC3708738

[10] Njuguna IN, Cranmer LM, Otieno VO, Mugo C, Okinyi HM, Benki-Nugent S, et al. Urgent versus post-stabilisation antiretroviral treatment in hospitalised HIV-infected children in Kenya (PUSH): a randomised controlled trial. Lancet HIV. 2018;5(1):e12–22. doi: 10.1016/S2352-3018(17)30167-4 29150377 PMC5777310

[11] Dahourou DL, Amorissani‐Folquet M, Yonaba C, Malateste K, Toni T, et al. Longitudinal cluster analysis of viral suppression during 25 months on antiretroviral therapy, adherence and factors associated in young West‐African children, in the MONOD ANRS 12206 cohort. 9th IAS Conf HIV Sci (2017). 2017.

[12] Amani-Bosse C, Dahourou DL, Malateste K, Amorissani-Folquet M, Coulibaly M, Dattez S, et al. Virological response and resistances over 12 months among HIV-infected children less than two years receiving first-line lopinavir/ritonavir-based antiretroviral therapy in Cote d’Ivoire and Burkina Faso: the MONOD ANRS 12206 cohort. J Int AIDS Soc. 2017;20(1):21362. doi: 10.7448/IAS.20.01.21362 28453240 PMC5515025

[13] Wrubel J, Moskowitz JT, Richards TA, Prakke H, Acree M, Folkman S. Pediatric adherence: perspectives of mothers of children with HIV. Soc Sci Med. 2005;61(11):2423–33. doi: 10.1016/j.socscimed.2005.04.034 15936134

[14] Haberer J, Mellins C. Pediatric adherence to HIV antiretroviral therapy. Curr HIV/AIDS Rep. 2009;6(4):194–200. doi: 10.1007/s11904-009-0026-8 19849962 PMC2967363

[15] Gross R, Bandason T, Langhaug L, Mujuru H, Lowenthal E, Ferrand R. Factors associated with self-reported adherence among adolescents on antiretroviral therapy in Zimbabwe. AIDS Care. 2015;27(3):322–6. doi: 10.1080/09540121.2014.969676 25338010 PMC4305494

[16] Nabukeera-Barungi N, Kalyesubula I, Kekitiinwa A, Byakika-Tusiime J, Musoke P. Adherence to antiretroviral therapy in children attending Mulago Hospital, Kampala. Ann Trop Paediatr. 2007;27(2):123–31. doi: 10.1179/146532807X192499 17565809

[17] Vreeman RC, Wiehe SE, Pearce EC, Nyandiko WM. A systematic review of pediatric adherence to antiretroviral therapy in low- and middle-income countries. Pediatr Infect Dis J. 2008;27(8):686–91. doi: 10.1097/INF.0b013e31816dd325 18574439

[18] Leroy V, Malateste K, Rabie H, Lumbiganon P, Ayaya S, Dicko F, et al. Outcomes of antiretroviral therapy in children in Asia and Africa: a comparative analysis of the IeDEA pediatric multiregional collaboration. J Acquir Immune Defic Syndr. 2013;62(2):208–19. doi: 10.1097/QAI.0b013e31827b70bf 23187940 PMC3556242

[19] Vreeman RC, Scanlon ML, Inui TS, McAteer CI, Fischer LJ, McHenry MS. Why did you not tell me?: perspectives of caregivers and children on the social environment surrounding child HIV disclosure in Kenya. LWW. 2015. 10.1097/QAD.0000000000000669 PMC8404532 26049538

[20] Vreeman RC, Gramelspacher AM, Gisore PO, Scanlon ML, Nyandiko WM. Disclosure of HIV status to children in resource-limited settings: a systematic review. J Int AIDS Soc. 2013;16(1):18466. doi: 10.7448/IAS.16.1.18466 23714198 PMC3665848

[21] Vreeman RC, Scanlon ML, Marete I, Mwangi A, Inui TS, McAteer CI, et al. Characteristics of HIV-infected adolescents enrolled in a disclosure intervention trial in western Kenya. AIDS Care. 2015;27 Suppl 1(sup1):6–17. doi: 10.1080/09540121.2015.1026307 26616121 PMC4685612

[22] Turissini ML, Nyandiko WM, Ayaya SO, Marete I, Mwangi A, Chemboi V, et al. The Prevalence of Disclosure of HIV Status to HIV-Infected Children in Western Kenya. J Pediatric Infect Dis Soc. 2013;2(2):136–43. doi: 10.1093/jpids/pit024 26619460 PMC11513781

[23] Montalto GJ, Sawe FK, Miruka A, Maswai J, Kiptoo I, Aoko A, et al. Diagnosis disclosure to adolescents living with HIV in rural Kenya improves antiretroviral therapy adherence and immunologic outcomes: A retrospective cohort study. PLoS One. 2017;12(10):e0183180. doi: 10.1371/journal.pone.0183180 28991913 PMC5633147

[24] Vreeman RC, McCoy BM, Lee S. Mental health challenges among adolescents living with HIV. J Int AIDS Soc. 2017;20(Suppl 3):21497. doi: 10.7448/IAS.20.4.21497 28530045 PMC5577712

[25] Magura J, Nhari SR, Nzimakwe TI. Barriers to ART adherence in sub-Saharan Africa: a scoping review toward achieving UNAIDS 95-95-95 targets. Front Public Health. 2025;13:1609743. doi: 10.3389/fpubh.2025.1609743 40556913 PMC12185539

[26] Smith R, Villanueva G, Probyn K, Sguassero Y, Ford N, Orrell C, et al. Accuracy of measures for antiretroviral adherence in people living with HIV. Cochrane Database Syst Rev. 2022;7(7):CD013080. doi: 10.1002/14651858.CD013080.pub2 35871531 PMC9309033

[27] Berg KM, Arnsten JH. Practical and conceptual challenges in measuring antiretroviral adherence. J Acquir Immune Defic Syndr. 2006;43 Suppl 1(Suppl 1):S79-87. doi: 10.1097/01.qai.0000248337.97814.66 17133207 PMC2866146

[28] Bangsberg DR. Less than 95% adherence to nonnucleoside reverse-transcriptase inhibitor therapy can lead to viral suppression. Clin Infect Dis. 2006;43(7):939–41. doi: 10.1086/507526 16941380

[29] Bangsberg DR, Charlebois ED, Grant RM, Holodniy M, Deeks SG, Perry S, et al. High levels of adherence do not prevent accumulation of HIV drug resistance mutations. AIDS. 2003;17(13):1925–32. doi: 10.1097/00002030-200309050-00011 12960825

[30] Bangsberg DR, Moss AR, Deeks SG. Paradoxes of adherence and drug resistance to HIV antiretroviral therapy. J Antimicrob Chemother. 2004;53(5):696–9. doi: 10.1093/jac/dkh162 15044425

[31] Leng X, Liang S, Ma Y, Dong Y, Kan W, Goan D, et al. HIV virological failure and drug resistance among injecting drug users receiving first-line ART in China. BMJ Open. 2014;4(10):e005886. doi: 10.1136/bmjopen-2014-005886 25319999 PMC4202012

[32] Adjé-Touré C, Hanson DL, Talla-Nzussouo N, Borget M-Y, Kouadio LY, Tossou O, et al. Virologic and immunologic response to antiretroviral therapy and predictors of HIV type 1 drug resistance in children receiving treatment in Abidjan, Côte d’Ivoire. AIDS Res Hum Retroviruses. 2008;24(7):911–7. doi: 10.1089/aid.2007.0264 18593341

[33] Vreeman RC, Wiehe SE, Ayaya SO, Musick BS, Nyandiko WM. Association of antiretroviral and clinic adherence with orphan status among HIV-infected children in Western Kenya. J Acquir Immune Defic Syndr. 2008;49(2):163–70. doi: 10.1097/QAI.0b013e318183a996 18769353

[34] Jittamala P, Puthanakit T, Chaiinseeard S, Sirisanthana V. Predictors of virologic failure and genotypic resistance mutation patterns in thai children receiving non-nucleoside reverse transcriptase inhibitor-based antiretroviral therapy. Pediatr Infect Dis J. 2009;28(9):826–30. doi: 10.1097/INF.0b013e3181a458f9 19654564

[35] Gody J-C, Charpentier C, Mbitikon O, Si-Mohamed A, LeGoff J, Grésenguet G, et al. High prevalence of antiretroviral drug resistance mutations in HIV-1 non-B subtype strains from African children receiving antiretroviral therapy regimen according to the 2006 revised WHO recommendations. J Acquir Immune Defic Syndr. 2008;49(5):566–9. doi: 10.1097/QAI.0b013e318183acae 19202461

[36] Hyatt JM, McKinnon PS, Zimmer GS, Schentag JJ. The Importance of Pharmacokinetic/Pharmacodynamic Surrogate Markers to Outcome. Clinical Pharmacokinetics. 1995;28(2):143–60. doi: 10.2165/00003088-199528020-00005 7736689

[37] Shuter J. Forgiveness of non-adherence to HIV-1 antiretroviral therapy. J Antimicrob Chemother. 2008;61(4):769–73. doi: 10.1093/jac/dkn020 18256112

[38] Tu W, Nyandiko WM, Liu H, Slaven JE, Scanlon ML, Ayaya SO, et al. Pharmacokinetics-based adherence measures for antiretroviral therapy in HIV-infected Kenyan children. J Int AIDS Soc. 2017;20(1):21157. doi: 10.7448/IAS.20.1.21157 28605170 PMC5515048

[39] Humphrey JM, Genberg BL, Keter A, Musick B, Apondi E, Gardner A, et al. Viral suppression among children and their caregivers living with HIV in western Kenya. J Int AIDS Soc. 2019;22(4):e25272. doi: 10.1002/jia2.25272 30983148 PMC6462809

[40] Edessa D, Sisay M, Asefa F. Second-line HIV treatment failure in sub-Saharan Africa: A systematic review and meta-analysis. PLoS One. 2019;14(7):e0220159. doi: 10.1371/journal.pone.0220159 31356613 PMC6663009

[41] Vreeman RC, Nyandiko WM, Liu H, Tu W, Scanlon ML, Slaven JE, et al. Measuring adherence to antiretroviral therapy in children and adolescents in western Kenya. J Int AIDS Soc. 2014;17(1):19227. doi: 10.7448/IAS.17.1.19227 25427633 PMC4245448

[42] Vreeman RC, Nyandiko WM, Liu H, Tu W, Scanlon ML, Slaven JE, et al. Comprehensive evaluation of caregiver-reported antiretroviral therapy adherence for HIV-infected children. AIDS Behav. 2015;19(4):626–34. doi: 10.1007/s10461-015-0998-x 25613594 PMC4393778

[43] Vreeman RC, Nyandiko WM, Liu H, Tu W, Scanlon ML, Slaven JE, et al. Measuring adherence to antiretroviral therapy in children and adolescents in western Kenya. J Int AIDS Soc. 2014;17(1):19227. doi: 10.7448/IAS.17.1.19227 25427633 PMC4245448

[44] Vreeman RC, Scanlon ML, Tu W, Slaven JE, McAteer CI, Kerr SJ, et al. Validation of a self-report adherence measurement tool among a multinational cohort of children living with HIV in Kenya, South Africa and Thailand. J Int AIDS Soc. 2019;22(5):e25304. doi: 10.1002/jia2.25304 31148372 PMC6543456

[45] Vreeman RC, Nyandiko WM, Ayaya SO, Walumbe EG, Inui TS. Cognitive interviewing for cross-cultural adaptation of pediatric antiretroviral therapy adherence measurement items. Int J Behav Med. 2014;21(1):186–96. doi: 10.1007/s12529-012-9283-9 23188670

[46] Tu W, Nyandiko WM, Liu H, Slaven JE, Scanlon ML, Ayaya SO, et al. Pharmacokinetics-based adherence measures for antiretroviral therapy in HIV-infected Kenyan children. J Int AIDS Soc. 2017;20(1):21157. doi: 10.7448/IAS.20.1.21157 28605170 PMC5515048

[47] Vreeman RC, Nyandiko WM, Liu H, Tu W, Scanlon ML, Slaven JE, et al. Measuring adherence to antiretroviral therapy in children and adolescents in western Kenya. J Int AIDS Soc. 2014;17(1):19227. doi: 10.7448/IAS.17.1.19227 25427633 PMC4245448

[48] WHO. HIV. www.who.int

[49] Kasper K, Moon B, Nguyen J, Orozco A, Chung K, Valencia J, et al. Homogeneous enzyme immunoassay for HIV-1 antiretroviral drugs: Darunavir and maraviroc. Stuttgart, Germany: International Association of Therapeutic Drug Monitoring & Clinical Toxicology Kultur-und Kongresszentrum Liederhalle. 2011.

[50] Holland DE-QR, Stefanski E, Moon B, Valdez J, Connor J. Rapid, automated immunoassay for therapeutic drug monitoring of nevirapine using ARK NVP-Test: method validation, application and comparison with HPLC plasma method. 45th Intersecience Conference on Antimicrobial Agents and Chemotheraphy (ICAAC); Washington, D.C, 2005.

[51] Cusato MVP, Bartoli A, Broglia M, Regazzi M. Comparison of an HPLC-UV method with a rapid automated immunoassay (EIA) to measure nevirapine concentration in HIV patients. 8th International Workshop on Clinical Pharmacology of HIV Theraphy: Budapest, Hungary, 2007.

[52] Nyandiko W, Holland S, Vreeman R, DeLong AK, Manne A, Novitsky V, et al. HIV-1 Treatment Failure, Drug Resistance, and Clinical Outcomes in Perinatally Infected Children and Adolescents Failing First-Line Antiretroviral Therapy in Western Kenya. J Acquir Immune Defic Syndr. 2022;89(2):231–9. doi: 10.1097/QAI.0000000000002850 34723922 PMC8752470

[53] Wood SN, Pya N, Säfken B. Smoothing Parameter and Model Selection for General Smooth Models. Journal of the American Statistical Association. 2016;111(516):1548–63.

[54] Wood SN. Fast Stable Restricted Maximum Likelihood and Marginal Likelihood Estimation of Semiparametric Generalized Linear Models. Journal of the Royal Statistical Society Series B: Statistical Methodology. 2010;73(1):3–36. doi: 10.1111/j.1467-9868.2010.00749.x

[55] Team RC. R: A language and environment for statistical computing. 2013.

[56] Vreeman RC, Ayaya SO, Musick BS, Yiannoutsos CT, Cohen CR, Nash D, et al. Adherence to antiretroviral therapy in a clinical cohort of HIV-infected children in East Africa. PLoS One. 2018;13(2):e0191848. doi: 10.1371/journal.pone.0191848 29466385 PMC5842873

[57] Kantor R, DeLong A, Schreier L, Reitsma M, Kemboi E, Orido M, et al. HIV-1 second-line failure and drug resistance at high-level and low-level viremia in Western Kenya. AIDS. 2018;32(17):2485–96. doi: 10.1097/QAD.0000000000001964 30134290 PMC6984814

[58] Saravanan S, Gomathi S, Delong A, Kausalya B, Sivamalar S, Poongulali S, et al. High discordance in blood and genital tract HIV-1 drug resistance in Indian women failing first-line therapy. J Antimicrob Chemother. 2018;73(8):2152–61. doi: 10.1093/jac/dky154 29800305 PMC6927885

[59] Godfrey C, Bobkova M, Boucher C, Ravasi G, Chen P, Zhang F, et al. Regional Challenges in the Prevention of Human Immunodeficiency Virus Drug Resistance. J Infect Dis. 2017;216(suppl_9):S816–9. doi: 10.1093/infdis/jix408 28968824 PMC5853282

[60] Meyers T, Sawry S, Wong JY, Moultrie H, Pinillos F, Fairlie L, et al. Virologic failure among children taking lopinavir/ritonavir-containing first-line antiretroviral therapy in South Africa. Pediatr Infect Dis J. 2015;34(2):175–9. doi: 10.1097/INF.0000000000000544 25741970 PMC4352713

[61] Carr A, Mackie NE, Paredes R, Ruxrungtham K. HIV drug resistance in the era of contemporary antiretroviral therapy: A clinical perspective. Antivir Ther. 2023;28(5):13596535231201162. doi: 10.1177/13596535231201162 37749751

[62] Vanangamudi M, Kurup S, Namasivayam V. Non-nucleoside reverse transcriptase inhibitors (NNRTIs): a brief overview of clinically approved drugs and combination regimens. Curr Opin Pharmacol. 2020;54:179–87. doi: 10.1016/j.coph.2020.10.009 33202360

[63] Waalewijn H, Turkova A, Rakhmanina N, Cressey TR, Penazzato M, Colbers A, et al. Optimizing pediatric dosing recommendations and treatment management of antiretroviral drugs using therapeutic drug monitoring data in Children Living With HIV. Ther Drug Monit. 2019;41(4):431–43. doi: 10.1097/FTD.0000000000000637 31008997 PMC6636807

[64] Siedner M, Moorhouse M, Simmons B, de Oliveira T, Lessells R, Giandhari J. Reduced efficacy of HIV-1 integrase inhibitors in patients with drug resistance mutations in reverse transcriptase. 2020. 10.1038/s41467-020-19801-x PMC7708638 33262331

[65] Sohn AH, Singtoroj T, Chokephaibulkit K, Lumbiganon P, Hansudewechakul R, Gani YM, et al. Long-Term Post-Transition outcomes of adolescents and young adults living with perinatally and non-perinatally acquired HIV in Southeast Asia. J Adolesc Health. 2023;72(3):471–9. doi: 10.1016/j.jadohealth.2022.10.021 36535867 PMC11772534

[66] Jegede OE, van Wyk B. Transition interventions for adolescents on antiretroviral therapy on transfer from pediatric to adult healthcare: a systematic review. Int J Environ Res Public Health. 2022;19(22):14911. doi: 10.3390/ijerph192214911 36429633 PMC9690836

