# Supplementary material for: Adherence to antiretroviral therapy and drug resistance impact evolving viral suppression among a cohort of children and adolescents living with perinatal HIV infection in western Kenya
Source: PLoS One. 2026 Sep 15;21(9):e0355492. doi: 10.1371/journal.pone.0355492 (PMC13577498; doi:10.1371/journal.pone.0355492)
Supplement: Supplementary file 1 [file pone.0355492.s001.docx]

**Supplemental Table 1. CAMP Adherence Questionnaire.**

| **Q#** | **Question** | **Response Options** |
| --- | --- | --- |
| **Q1** | Who gives the participant his/her medicines? | Mother, Father, Grandparent, Auntie/Uncle, Guardian, Relative who lives in the home, relative who lives outside of the home, neighbor, sibling, house help, child takes med themselves, other |
| **Q2*** | Does the child know that he/she is taking the medicines for HIV? | Yes, no, don’t know, refused (yes +1) |
| **Q3** | How many people in your household take medicines for HIV? | Open text, don’t know, refused |
| **Q4*** | Do you ever have problems keeping time with the medicines? If so, when? | Yes, no, don’t know, refused (yes +1) |
| **Q4a** |  | Mornings, evenings, weekends, weekdays, other |
| **Q5*** | Do you ever have problems with getting your child to take the medicines? | Yes, no, don’t know, refused (yes +1) |
| **Q5a** | If yes, what problems? |  |
| **Q6** | Some families tell us that their child worries them or makes it difficult to give them medicines. Has your child not taken medicines for any of these reasons? | He/she does not know why taking the medicines or keeps asking questions about the medicines; He/she forgot to take medicine; He/she felt ill or was vomiting; He/she was playing or at school or work; He/she refused to take medicine; He/she has problems with 1 formulation (tablets, liquids) ; He/she finds medicines too bitter; He/she can’t take medicines without food; Other |
| **Q7** | Sometimes, a child does not take their medicines every day or at the same time every day because of difficulties for the caregiver. I am going to read a list of issues that may be problems for you as a caregiver in having the child take the medicines. Tell me when you hear a problem mentioned that applies to you or the child’s caregiver: | I was away from home (work, field, etc.); I had difficulty with the instructions for the medicines; I was not always around with the child; I was too busy and forgot; I did not want others to see; I had trouble with timing or giving the doses on time; None of the above |
| **Q8** | Sometimes, problems at the clinic make it difficult for families to give these medicines every day. Have any of these things been a problem for you: | There was no money to purchase medicine (if not offered at AMPATH); The medicine was not available in the pharmacy; We finished or ran out of the medicines; None of the above |
| **Q9*** | In the past week: On how many days did the child miss at least one dose? | (any value >0, +1) |
| **Q10*** | On how many days did the child take a dose more than an hour late? | (any value >0, +1) |
| **Q11** | How many extra doses or syringes of medicine did the child take? | Open text |
| **Q12*** | How many doses of medicine has your child missed in the last month? | (any value >0, +1) |

### **Footnotes**: Questions marked with an asterisk (*) contributed one point each to the composite non-adherence score; higher scores indicate greater non-adherence. For starred questions, responses of “Yes” or any numeric value greater than zero were coded as non-adherent. Responses of “Don’t know” or “Refused” were coded as missing. Questionnaire items were used to derive adherence summary variables presented in Table 2.
