# Supplementary material for: Adherence to antiretroviral therapy and drug resistance impact evolving viral suppression among a cohort of children and adolescents living with perinatal HIV infection in western Kenya
Source: PLoS One. 2026 Sep 15;21(9):e0355492. doi: 10.1371/journal.pone.0355492 (PMC13577498; doi:10.1371/journal.pone.0355492)
Supplement: Supplementary file 2 [file pone.0355492.s002.docx]

#### Supplemental Table 2. Characteristics of participants at CAMP enrollment and Blood Draw 1, stratified by number of blood draws completed.

| **Variable** | **Total**  **(N = 692)** | **Two BDs**  **(N = 328)** | **One BD**  **(N = 364)** | **p-value** |
| --- | --- | --- | --- | --- |
| **Months between CAMP enrollment and BD1, mean (SD)** | 2.0 (1.0) | 1.8 (1.0) | 2.2 (1.0) | <0.001 |
| Missing | 27 | 19 | 8 |  |
| **Age at CAMP enrollment (years), mean (SD)** | 8.6 (3.3) | 8.5 (3.4) | 8.6 (3.3) | 0.486 |
| **Age group at CAMP enrollment** |  |  |  | 0.445 |
| <5 years | 126 (18) | 57 (17) | 69 (19) |  |
| ≥5 to <10 years | 341 (49) | 170 (52) | 171 (47) |  |
| ≥10 years | 225 (33) | 101 (31) | 124 (34) |  |
| **Gender** |  |  |  | 0.068 |
| Female | 356 (51) | 181 (55) | 175 (48) |  |
| Male | 336 (49) | 147 (45) | 189 (52) |  |
| **Orphan status at CAMP enrollment** |  |  |  | <0.001 |
| Double orphan | 87 (13) | 53 (16) | 34 (9) |  |
| Maternal orphan | 75 (11) | 45 (14) | 30 (8) |  |
| Paternal orphan | 72 (10) | 44 (13) | 28 (8) |  |
| Non-orphan | 457 (66) | 186 (57) | 271 (74) |  |
| Missing | 1 (0) | 0 (0) | 1 (0) |  |
| **Years on ART at BD1, mean (SD)** | 2.8 (2.0) | 2.6 (1.9) | 3.0 (2.0) | 0.011 |
| Missing | 2 | 0 | 2 |  |

**Footnotes**: Unless otherwise noted all results are presented as N (%). *P-values compare participants with two blood draws versus one blood draw.*

***Abbreviations****: BD1, Blood Draw 1; SD, Standard Deviation.*
