# Supplementary material for: Adherence to antiretroviral therapy and drug resistance impact evolving viral suppression among a cohort of children and adolescents living with perinatal HIV infection in western Kenya
Source: PLoS One. 2026 Sep 15;21(9):e0355492. doi: 10.1371/journal.pone.0355492 (PMC13577498; doi:10.1371/journal.pone.0355492)
Supplement: Supplementary file 3 [file pone.0355492.s003.docx]

Supplemental Table 3. Comparison of participants with and without viral load (VL) results at blood draw 1 (BD1)

| **Variable** | **Total**  **(N = 692)** | **No VL Measure (N = 228)** | **Has VL Measure**  **(N = 464)** | **p-value** |
| --- | --- | --- | --- | --- |
| **Months between CAMP enrollment and BD1, mean (SD)** | 2.0 (1.0) | 2.5 (0.9) | 1.7 (1.0) | <0.001 |
| Missing | 27 | 4 | 23 |  |
| **Age at CAMP enrollment (years), mean (SD)** | 8.6 (3.3) | 8.9 (3.3) | 8.4 (3.3) | 0.060 |
| **Gender** |  |  |  | 0.518 |
| Female | 356 (51) | 113 (50) | 243 (52) |  |
| Male | 336 (49) | 115 (50) | 221 (48) |  |
| **Orphan status at CAMP enrollment** |  |  |  | 0.457 |
| Double orphan | 87 (13) | 28 (12) | 59 (13) |  |
| Maternal orphan | 75 (11) | 27 (12) | 48 (10) |  |
| Paternal orphan | 72 (10) | 19 (8) | 53 (11) |  |
| Non-orphan | 457 (66) | 153 (67) | 304 (66) |  |
| Missing | 1 (0) | 1 (0) | 0 (0) |  |
| **Years on ART at BD1, mean (SD)** | 2.8 (2.0) | 2.9 (2.1) | 2.7 (1.9) | 0.434 |
| Missing | 2 | 2 | 0 |  |

**Footnote**: Unless otherwise noted all results are presented as N (%). *P-values compare participants with two blood draws versus one blood draw.*

**Abbreviations:** BD1, blood draw 1; VL, viral load; ART, antiretroviral therapy; SD, Standard Deviation.
