# Supplementary material for: Adherence to antiretroviral therapy and drug resistance impact evolving viral suppression among a cohort of children and adolescents living with perinatal HIV infection in western Kenya
Source: PLoS One. 2026 Sep 15;21(9):e0355492. doi: 10.1371/journal.pone.0355492 (PMC13577498; doi:10.1371/journal.pone.0355492)
Supplement: Supplementary file 4 [file pone.0355492.s004.docx]

Supplemental Table 4. Bivariate summaries of model covariates used in the generalized additive model for treatment failure at Blood Draw 1, stratified by viral load outcome.

| **Variable** | **Total** | **VL ≤ 1,000** | **VL > 1,000** |
| --- | --- | --- | --- |
| **% adherence using MEMS (z-score, enrollment to BD1), mean (SD)** | 1.2 (1.0) | 1.4 (0.9) | 1.0 (1.0) |
| **Number treatment interruptions >48 hours standardized by number of weeks in period (enrollment to BD1) , mean (SD)** | 0.1 (0.2) | 0.1 (0.2) | 0.1 (0.3) |
| **Duration (hours) of maximum treatment interruption (log10, enrollment to BD1) , mean (SD)** | 1.5 (0.4) | 1.5 (0.4) | 1.6 (0.5) |
| **At least 1 treatment interruption >48 hours (enrollment to BD1) , mean (SD)** | 0.3 (0.5) | 0.2 (0.4) | 0.4 (0.5) |
| **Adherence questionnaire score at enrollment, mean (SD)** | 1.0 (1.2) | 0.9 (1.2) | 1.3 (1.4) |
| **Adherence questionnaire score at BD1, mean (SD)** | 0.9 (1.0) | 0.8 (1.0) | 0.9 (1.1) |
| **Drug levels** |  |  |  |
| Sub-therapeutic | 46 (13) | 32 (13) | 14 (13) |
| Supra-therapeutic | 163 (47) | 119 (50) | 44 (42) |
| Therapeutic | 136 (39) | 89 (37) | 47 (45) |
| **Gender** |  |  |  |
| Female | 186 (54) | 132 (55) | 54 (51) |
| Male | 159 (46) | 108 (45) | 51 (49) |
| **log10 years on ART, mean (SD)** | 0.3 (0.4) | 0.3 (0.4) | 0.3 (0.4) |
| **Age (years) , mean (SD)** | 8.4 (3.2) | 8.6 (3.2) | 7.9 (3.2) |
| **Orphan status at enrollment** |  |  |  |
| Double orphan | 48 (14) | 33 (14) | 15 (14) |
| Maternal orphan | 35 (10) | 29 (12) | 6 (6) |
| Non-orphan | 218 (63) | 149 (62) | 69 (66) |
| Paternal orphan | 44 (13) | 29 (12) | 15 (14) |

**Footnote**: Unless otherwise noted all results are presented as N (%).

**Abbreviations:** BD1, blood draw 1; BD2, blood draw 2; VL, viral load; ART, antiretroviral therapy; SD, Standard Deviation.
