# Supplementary material for: Adherence to antiretroviral therapy and drug resistance impact evolving viral suppression among a cohort of children and adolescents living with perinatal HIV infection in western Kenya
Source: PLoS One. 2026 Sep 15;21(9):e0355492. doi: 10.1371/journal.pone.0355492 (PMC13577498; doi:10.1371/journal.pone.0355492)
Supplement: Supplementary file 5 [file pone.0355492.s005.docx]

### Supplemental Table 5. Bivariate summaries of model covariates stratified by number of drug resistance mutations at Blood Draw 1

| **Variable** | **Total** | **0–3 DRMs** | **4–7 DRMs** | **8–12 DRMs** |
| --- | --- | --- | --- | --- |
| **% adherence using MEMS (z-score, enrollment to BD1), mean (SD)** | 0.9 (1.1) | 0.6 (1.1) | 1.0 (1.0) | 1.4 (1.2) |
| **Number treatment interruptions >48 hours standardized by number of weeks in period (enrollment to BD1) , mean (SD)** | 0.2 (0.3) | 0.3 (0.4) | 0.1 (0.2) | 0.2 (0.3) |
| **Duration (hours) of maximum treatment interruption (log10, enrollment to BD1) , mean (SD)** | 1.6 (0.5) | 1.8 (0.5) | 1.6 (0.5) | 1.6 (0.5) |
| **At least 1 treatment interruption >48 hours (enrollment to BD1) , mean (SD)** | 0.4 (0.5) | 0.5 (0.5) | 0.4 (0.5) | 0.5 (0.5) |
| **Adherence questionnaire score at enrollment, mean (SD)** | 1.3 (1.4) | 1.8 (1.8) | 1.2 (1.3) | 1.0 (1.0) |
| **Adherence questionnaire score at BD1, mean (SD)** | 1.0 (1.1) | 1.2 (1.4) | 0.9 (0.9) | 1.1 (1.0) |
| **Drug levels** |  |  |  |  |
| Sub-therapeutic | 12 (13) | 7 (30) | 5 (9) | 0 (0) |
| Supra-therapeutic | 37 (41) | 3 (13) | 26 (46) | 8 (73) |
| Therapeutic | 42 (46) | 13 (57) | 26 (46) | 3 (27) |
| **Gender** |  |  |  |  |
| Female | 46 (51) | 13 (57) | 28 (49) | 5 (45) |
| Male | 45 (49) | 10 (43) | 29 (51) | 6 (55) |
| **log10 years on ART, mean (SD)** | 0.4 (0.4) | 0.4 (0.4) | 0.3 (0.4) | 0.7 (0.2) |
| **Age (years) , mean (SD)** | 8.1 (3.0) | 8.1 (3.6) | 8.1 (2.9) | 8.1 (2.5) |
| **Orphan status at enrollment** |  |  |  |  |
| Double orphan | 14 (15) | 2 (9) | 10 (18) | 2 (18) |
| Maternal orphan | 4 (4) | 2 (9) | 0 (0) | 2 (18) |
| Non-orphan | 60 (66) | 13 (57) | 42 (74) | 5 (45) |
| Paternal orphan | 13 (14) | 6 (26) | 5 (9) | 2 (18) |
| **Log10 viral load at BD1, mean (SD)** | 4.0 (0.7) | 3.6 (0.6) | 4.1 (0.7) | 4.2 (0.8) |

**Footnote**: Unless otherwise noted all results are presented as N (%).

**Abbreviations:** BD1, blood draw 1; BD2, blood draw 2; VL, viral load; ART, antiretroviral therapy; SD, Standard Deviation.
