# Supplementary material for: Adherence to antiretroviral therapy and drug resistance impact evolving viral suppression among a cohort of children and adolescents living with perinatal HIV infection in western Kenya
Source: PLoS One. 2026 Sep 15;21(9):e0355492. doi: 10.1371/journal.pone.0355492 (PMC13577498; doi:10.1371/journal.pone.0355492)
Supplement: Supplementary file 6 [file pone.0355492.s006.docx]

### Supplemental Table 6. Bivariate summaries of model covariates stratified by viral load suppression at Blood Draw 2

| **Variable** | **Total** | **VL ≤1000** | **VL >1000** |
| --- | --- | --- | --- |
| **% adherence using MEMS (z-score, BD1 to BD2), mean (SD)** | 1.5 (0.7) | 1.6 (0.7) | 1.4 (0.7) |
| **Number treatment interruptions >48 hours standardized by number of weeks in period (BD1 to BD2) , mean (SD)** | 0.0 (0.1) | 0.0 (0.1) | 0.1 (0.1) |
| **At least 1 treatment interruption >48 hours (BD1 to BD2), mean (SD)** | 0.8 (0.4) | 0.8 (0.4) | 0.7 (0.5) |
| **Adherence questionnaire score at BD1, mean (SD)** | 1.0 (1.2) | 1.0 (1.1) | 1.1 (1.3) |
| **Adherence questionnaire score at BD2, mean (SD)** | 1.3 (1.2) | 1.5 (1.3) | 1.0 (1.0) |
| **Drug levels (BD2)** |  |  |  |
| Sub-therapeutic | 9 (16) | 6 (16) | 3 (15) |
| Supra-therapeutic | 30 (53) | 18 (49) | 12 (60) |
| Therapeutic | 18 (32) | 13 (35) | 5 (25) |
| **Gender** |  |  |  |
| Female | 29 (51) | 20 (54) | 9 (45) |
| Male | 28 (49) | 17 (46) | 11 (55) |
| **log10 years on ART, mean (SD)** | 0.3 (0.4) | 0.3 (0.4) | 0.2 (0.4) |
| **Age (years), mean (SD)** | 7.6 (3.2) | 7.4 (3.4) | 7.9 (2.9) |
| **Orphan status at enrollment** |  |  |  |
| Double orphan | 10 (18) | 6 (16) | 4 (20) |
| Non-orphan | 34 (60) | 23 (62) | 11 (55) |
| Single orphan | 13 (23) | 8 (22) | 5 (25) |
| **Log10 viral load at BD2, mean (SD)** | 4.1 (0.7) | 4.1 (0.8) | 4.2 (0.6) |

**Footnote**: Unless otherwise noted all results are presented as N (%).

**Abbreviations:** BD1, blood draw 1; BD2, blood draw 2; VL, viral load; ART, antiretroviral therapy; SD, Standard Deviation.
