# Supplementary material for: Adherence to antiretroviral therapy and drug resistance impact evolving viral suppression among a cohort of children and adolescents living with perinatal HIV infection in western Kenya
Source: PLoS One. 2026 Sep 15;21(9):e0355492. doi: 10.1371/journal.pone.0355492 (PMC13577498; doi:10.1371/journal.pone.0355492)
Supplement: Supplementary file 7 [file pone.0355492.s007.docx]

**Appendix A. Transformation of % adherence using MEMS**

The untransformed observed % MEMS adherence data were highly skewed to the right (Figure 1, left). To allow for more granularity in the upper portion of the distribution, we transformed the data to a z-score (Figure 1, right). To perform the transformation, we first modified the observed data (which had some values exactly 0 and 1), to be strictly between 0 and 1 by adding 1 and dividing by 102. These values were then transformed to a standard normal z-score distribution as shown in Figure 2. This transformation results in a value of 0.5 being mapped to 0; 0.98 to approximately 2 and 0.02 to approximately -2.

Figure 1. Histogram of the untransformed percent Adherence using MEMS (left plot) and histogram of the transformed percent Adherence using MEMS (right plot).

Figure 2. Plot of z-score transformation, showing how a % adherence using MEMS measurement was transformed to a standard normal z-score.
