# Supplementary material for: Adherence to antiretroviral therapy and drug resistance impact evolving viral suppression among a cohort of children and adolescents living with perinatal HIV infection in western Kenya
Source: PLoS One. 2026 Sep 15;21(9):e0355492. doi: 10.1371/journal.pone.0355492 (PMC13577498; doi:10.1371/journal.pone.0355492)
Supplement: Peer review history

# Peer review: Decision Letter 0

**DOI:** 10.1371/journal.pone.0355492.r001

**Authors:** YAGAI BOUBA

23 Nov 2025

Dear Dr. Vreeman,

Thank you for submitting your manuscript to PLOS ONE. After careful consideration, we feel that it has merit but does not fully meet PLOS ONE’s publication criteria as it currently stands. Therefore, we invite you to submit a revised version of the manuscript that addresses the points raised during the review process.

**major revision** , and the authors should carefully address **all reviewer comments**  as well as the detailed editorial points below, distinguishing required changes from recommended improvements in their response.

Please submit your revised manuscript by Jan 07 2026 11:59PM. If you will need more time than this to complete your revisions, please reply to this message or contact the journal office at plosone@plos.org. When you're ready to submit your revision, log on to https://www.editorialmanager.com/pone/ and select the 'Submissions Needing Revision' folder to locate your manuscript file.

- A rebuttal letter that responds to each point raised by the academic editor and reviewer(s). You should upload this letter as a separate file labeled 'Response to Reviewers'.
- A marked-up copy of your manuscript that highlights changes made to the original version. You should upload this as a separate file labeled 'Revised Manuscript with Track Changes'.
- An unmarked version of your revised paper without tracked changes. You should upload this as a separate file labeled 'Manuscript'.

If you would like to make changes to your financial disclosure, please include your updated statement in your cover letter. Guidelines for resubmitting your figure files are available below the reviewer comments at the end of this letter.

If applicable, we recommend that you deposit your laboratory protocols in protocols.io to enhance the reproducibility of your results. Protocols.io assigns your protocol its own identifier (DOI) so that it can be cited independently in the future. For instructions see: https://journals.plos.org/plosone/s/submission-guidelines#loc-laboratory-protocols. Additionally, PLOS ONE offers an option for publishing peer-reviewed Lab Protocol articles, which describe protocols hosted on protocols.io. Read more information on sharing protocols at https://plos.org/protocols?utm_medium=editorial-email&utm_source=authorletters&utm_campaign=protocols.

We look forward to receiving your revised manuscript.

Kind regards,

YAGAI BOUBA, PhD

Academic Editor

PLOS ONE

Journal Requirements:

When submitting your revision, we need you to address these additional requirements.

1. Please ensure that your manuscript meets PLOS ONE's style requirements, including those for file naming. The PLOS ONE style templates can be found at

https://journals.plos.org/plosone/s/file?id=wjVg/PLOSOne_formatting_sample_main_body.pdf and

https://journals.plos.org/plosone/s/file?id=ba62/PLOSOne_formatting_sample_title_authors_affiliations.pdf

2. Thank you for stating the following financial disclosure:

Funding for this project was to RV and RK from the National Institutes of Allergy and

Infectious Diseases at the National Institutes of Health [grant number: R01 AI120792]. The NIH had nothing to do with study design, data collection, analysis, decision to publish, or preparation of the manuscript.

Please state what role the funders took in the study. If the funders had no role, please state: "The funders had no role in study design, data collection and analysis, decision to publish, or preparation of the manuscript."

If this statement is not correct you must amend it as needed.

Please include this amended Role of Funder statement in your cover letter; we will change the online submission form on your behalf.

3. We notice that your supplementary figures and tables are uploaded with the file type 'Figure'. Please amend the file type to 'Supporting Information'. Please ensure that each Supporting Information file has a legend listed in the manuscript after the references list.

4. Please amend your list of authors on the manuscript to ensure that each author is linked to an affiliation. Authors’ affiliations should reflect the institution where the work was done (if authors moved subsequently, you can also list the new affiliation stating “current affiliation:….” as necessary).

5. Please amend the manuscript submission data (via Edit Submission) to include author J Aluoch and R Kantor

6. Please amend your authorship list in your manuscript file to include author Josephine Alouch

7. One of the noted authors is a group or consortium [for the RESPECT (RESistance in a PEdiatric CohorT) Study]. In addition to naming the author group, please list the individual authors and affiliations within this group in the acknowledgments section of your manuscript. Please also indicate clearly a lead author for this group along with a contact email address.

8. Please upload a copy of Figure 3a and 3b, to which you refer in your text on pages 19 and 22. If the figure is no longer to be included as part of the submission please remove all reference to it within the text.

9. If the reviewer comments include a recommendation to cite specific previously published works, please review and evaluate these publications to determine whether they are relevant and should be cited. There is no requirement to cite these works unless the editor has indicated otherwise.

Additional Editor Comments:

The study seeks to evaluate how patterns of ART adherence in children and adolescents influence treatment failure and HIV drug resistance development. The paper is globally well written, with appropriate statistical approach. However, to fully achieve relevance and scientific clarity, major revisions are needed to address some gaps, clarify study procedures, and explicitly interpret the findings within the current ART landscape. Plesse consider the following points:

TITLE:

1. I would suggest the title to reflect the study population because it includes both children and adolescents (10-14 yrs). Although you have a well characterized cohort, I don't have the impresion considering the follow up time that using the word "long-term" in the title is correct.

ABSTRACT

2. As explained in comment 1 below, please state specific objective.

As explained in the methods secton below, clarify this issues of defining treatment failure as viral load (VL) >1,000 copies/mL, ensure consistency with guidelines

For DR, indicate the gene and the sequencing methods

The design study should be clear, indicating study periods.

Revise conclusion with respect to the clearly specified objectives.

INTRODUCTION

3. The current objective is presented broadly and does not clearly reflect the analytical strategy described later in the manuscript.

Please revise the Introduction to state a precise and focused primary objective (and secondary objectives if applicable) that focus on this stage of the study.

4. I suggest you strengthen the rationale with: Why these historical samples (collected in 2010–2013) are relevant today and how the combination of MEMS, plasma drug levels, and treatment interruption measures adds value beyond existing evidence.

METHODS

5. There is substantial repetition across “Study Setting,” “Study Design,” “CAMP study procedures,” and “Sample procedures.”

Examples include: repeating the eligibility criteria, repeating VL/genotyping testing descriptions and others

Provide complete details on sample storage and handling. Because all plasma samples were collected between 2010–2013 and tested years later under new funding, it is essential to document:

• storage temperature (−80°C???),

• storage duration

• freezer monitoring and quality assurance measures,

• whether any samples experienced freeze–thaw cycles

• how stored samples were managed prior to testing.

These details are needed to interpret the validity of VL, DRM, and drug-level assays.

6. Provide Timeline for Laboratory Testing. The manuscript states that testing occurred after new funding but does not specify when.

Please indicate the period during which VL, drug levels, and genotyping were performed. In addition, indicate the assay failure rate of each test, this will an idea about the sample integrity.

Ensure transparency in reporting by stating for example whether storage conditions or RNA degradation played a role.

7. The authors used several methods to measure adherence. While this might be useful, expand the Rationale for Multiple Adherence Measures.

8. Streamline Redundant Sections between Study Setting, Study Design, and Original CAMP Procedures, and report especially relevant information directly to this study.

RESULTS

9. Clarify the use of standard terminology throughout the paper.

For viral load outcomes, ensure correct usage:

• “Suppressed” = <1000 copies/mL???

• “Undetectable” = <40 copies/mL???

TF is mistakenly taken for viral non suppression in some sentences. Please ensure consistency with known definition in the field (WHO or national guidelines).

10. Tables and figures: Overall, the tables and figures require clearer labelling, consistent variable naming, explicit definitions of numerical values and abbreviations, improved legends, and removal of ambiguous or unexplained formatting elements. These changes are essential to ensure that readers can correctly interpret the results and that the presentation aligns with the variables and methods described in the manuscript.

Specifically:

- Improve clarity and completeness of row and variable labels

- Many tables contain missing, incomplete, or ambiguous row names (e.g., “value,” blank rows, or unclear variable titles).

- All row labels should fully correspond to the variables defined in the Methods (e.g., MEMS %, number of >48h interruptions, BD1/BD2 VL, etc.).

- Clearly indicate what numerical values represent

Across several tables, it is unclear whether the numbers indicate counts (n), percentages, means (SD), or effect measures (OR/RR with CI).

Column headers or footnotes must explicitly define each type of value.

- Standardize use of parentheses and statistical notation

Bracketed values appear without explanation in some tables (particularly Table 3).

Every parenthetical value (SD, IQR, 95% CI, transformed variable) should be clearly identified.

- Ensure consistent and descriptive variable naming

Some variables appear truncated or unclear (“value???” or inconsistent acronyms).

All variables should be fully spelled out and consistent with terminology used elsewhere in the manuscript.

- All tables should include a footnote defining abbreviations (e.g., BD1, BD2, TF, DRM, VL, MEMS).

- Effect estimates/confidence intervals, and p-values should appear in separate columns to improve readability.

- The row labeled “Log10 VL at BD1 (see Figure 3b)” should be renamed simply “Log10 VL at BD1”.

Any cross-referencing should be placed in the caption, not inside the table body.

Discussions:

11. Deepen Interpretation of Interruption Patterns (NNRTI pharmacokinetic profiles???, Selective drug pressure dynamics???, etc....)

12. Explicitly Translate Findings to Today’s DTG Era, this is a critical revision to see the implications of your results in current ART era. In particular, please include a clear discussion on:

- NNRTI-Specific Findings (Patterns of EFV/NVP resistance, NNRTI-associated adherence thresholds????)

- Relevance to DTG-Based Regimens (in particular, how historical NRTI/NNRTI resistance affects modern DTG backbones)

- Relevance for Adolescents Transitioning From NNRTIs to DTG

This section is essential for framing the manuscript’s modern clinical value.

13. Extend the limitations with long-term sample storage, lack of contemporaneous laboratory testing, and missing data patterns and implications for bias.

CONCLUSIONS

14. Revise the Conclusion to Match Revised Objectives, after clarifying objectives (Highlight the key insights that remain relevant in today’s ART landscape).

[Note: HTML markup is below. Please do not edit.]

Reviewers' comments:

Reviewer's Responses to Questions

**Comments to the Author**

1. Is the manuscript technically sound, and do the data support the conclusions?

Reviewer #1: Partly

**********

2. Has the statistical analysis been performed appropriately and rigorously?

Reviewer #1: Yes

**********

3. Have the authors made all data underlying the findings in their manuscript fully available?

The PLOS Data policy

Reviewer #1: Yes

**********

4. Is the manuscript presented in an intelligible fashion and written in standard English?

Reviewer #1: Yes

**********

Reviewer #1: General comments

The authors presented an in-depth analysis of the relationship between ART drug adherence, treatment failure, and HIV drug resistance. The study is well-conceived and has the potential to make a meaningful contribution to the field. However, several aspects of the manuscript require major revisions to enhance its clarity, relevance, and impact. First, the data used in the study appear to be from an earlier treatment era when NNRTIs were commonly prescribed. While the authors acknowledge this, the manuscript does not sufficiently emphasize the relevance of these findings in the current context of evolving HIV treatment guidelines. The authors can elaborate more on that in the discussion section. I would also suggest that the authors revise the flow of the Introduction and Methods sections for clarity and coherence. Please find below my detailed feedback.

Additionally, I recommend the authors to:

• Describe the figure legends; figures have only the title, and legends should increase the compression of the study figures

• Improve the tables to meet the standard formatting and clarity expected in high-quality scientific manuscripts

• Double check the entire manuscript to ensure that the full spellings of most of the acronyms are not presented many times (e.g. TF, Blood draw 1 & 2, EFV, NVP…)

Specific comments

Title:

1. The title mentioned only children with perinatal infections, but the authors also included adolescents aged 10-14 years. Update the title to reflect the exact target population

2. The mean time on treatment is less than 3 years; using “long-term” to describe the cohort does not seem appropriate

Abstract:

3. The authors mentioned that the main objective was to demonstrate how and when non-adherence to ART leads to treatment failure and HIV drug resistance, but after going through the content, it’s not clear if the aim was achieved. The conclusion is vague.

Introduction

4. I suggest the authors to revise the entire introduction to have a better flow. They may also remove some information that are not relevant for the current paper (e.g. lines 29-37) and avoid redundancy.

Methods:

5. Redundant information in the study design and study settings. Either combine the two sections or only present the settings in the first section and the eligibility criteria plus the study site in the second section (design)

6. Line 104 “participants received routine AMPATH clinical examinations”, provide more details on that particular clinical examinations

7. It is unclear why two phases of enrollment. What is their importance for the study design? Authors should also mention the number of participants enrolled in the first phase

8. Instead of presenting the full methodology of the original study design before presenting the analysis done as part of the current manuscript (lines 135 to 141), the manuscript will gain in clarity if the authors mention the main study with its reference, then present the methods of the current nested analysis

Results:

9. Table 1 should be better described. For the first row, for example, it’s unclear what the data presented means

10. In the methods section, those with only BD1 were the ones from the second phase, but in the results section (lines 276-280), the authors mentioned they were from the first phase.

11. A dot is missing between the parentheses and the beginning of the next sentence with “Although…” (line 280)

12. Undetectable is less than 40 copies/mL and not 1000 copies/mL as mentioned by the authors (line 297). Suppressed VL would be more appropriate.

13. Why the authors concluded that age was also associated with TF despite the fact that the CI of the OR included 1? Also, I would suggest the authors to explicitly mention whether the variable reduce or increase the odd of TF instead of just saying variable X was associated.

14. Table 4a could be better presented, maybe by adding another column for the p-value. It would also be interesting to have the proportions (TF vs non-TF and DRM vs non-DRM) for each variable to better interpret the results. Same for table 4b. Authors should also consider merging tables 4a and 4b into one, with three main columns for TF at BD1, TF at BD2 and DRM. Within each column, they may create sub-columns to add proportions and separate OR (CI) from p-values.

Discussion

15. The authors mentioned that “there have been few pediatric studies evaluating the impact of drug resistance on viral failure for children living with HIV” (lines 374-375) which is not really true since there have been a lot of studies in low-income settings generating evidence of drug resistance among children and adolescents over the past decade. Authors may consider acknowledging that and citing a few of them, and maybe mention the fact that most of the studies are still focus on adults

16. The authors mentioned that “Using this strategy, we found a complex relationship, where among those with shorter interruptions (<48 hours), the longer the interruption in that window up to 48-hours, the more DR mutations the participant had. For those with longer interruptions (>48-hours), and even longer duration was protective against DR mutations.”. What could be the potential explanations? Do you really think the longer duration off-treatment was “protective against developing HIVDR mutations” or is it due to the fact that among those with less or no exposure to ART, viruses reverted to wild-type because they are more competent for replication?

17. What is the added value of your research in the current context? Now that most of the people (including children and adolescents) living with HIV have been transitioned to DTG-based treatment, how your study can contribute in this era? What lessons should we learn so that we do not make the same mistakes with DTG? I will suggest the authors to further discuss this to ensure the relevance of their research.

**********

what does this mean?). If published, this will include your full peer review and any attached files.

If you choose “no”, your identity will remain anonymous but your review may still be made public.

**Do you want your identity to be public for this peer review?**  For information about this choice, including consent withdrawal, please see our Privacy Policy

Reviewer #1: No

**********

[NOTE: If reviewer comments were submitted as an attachment file, they will be attached to this email and accessible via the submission site. Please log into your account, locate the manuscript record, and check for the action link "View Attachments". If this link does not appear, there are no attachment files.]

To ensure your figures meet our technical requirements, please review our figure guidelines: https://journals.plos.org/plosone/s/figures

You may also use PLOS’s free figure tool, NAAS, to help you prepare publication quality figures: https://journals.plos.org/plosone/s/figures#loc-tools-for-figure-preparation.

NAAS will assess whether your figures meet our technical requirements by comparing each figure against our figure specifications.

# Author comment: Author response to Decision Letter 1

**DOI:** 10.1371/journal.pone.0355492.r002

16 Jun 2026

Dear Editorial Team and Reviewers,

Thank you for this opportunity to revise and resubmit our manuscript. Please find below point-by-point responses to each of the (in bold) editorial points and reviewer’s comments. All edits made are also indicated as track changes in the manuscript.

We hope that you will find this revised manuscript suitable for publication in PLOS One.

Editorial Journal Requirements:

Please ensure that your manuscript meets PLOS ONE's style requirements, including those for file naming.

We have made careful revisions to ensure that all style requirements, including for file naming are correct.

Please state what role the funders took in the study. If the funders had no role, please state: "The funders had no role in study design, data collection and analysis, decision to publish, or preparation of the manuscript." If this statement is not correct you must amend it as needed. Please include this amended Role of Funder statement in your cover letter; we will change the online submission form on your behalf.

The funders indeed had no role, and we have amended the Role of Funder statement in the cover letter to the requested phrasing.

We notice that your supplementary figures and tables are uploaded with the file type 'Figure'. Please amend the file type to 'Supporting Information'. Please ensure that each Supporting Information file has a legend listed in the manuscript after the references list.

These changes have now been made.

Please amend your list of authors on the manuscript to ensure that each author is linked to an affiliation. Authors’ affiliations should reflect the institution where the work was done (if authors moved subsequently, you can also list the new affiliation stating “current affiliation:….” as necessary).

We have amended the author affiliations. Dr. Michael Scanlon’s affiliations now reflect the institution where the work was done (Icahn School of Medicine at Mount Sinai) and his current affiliation (DePauw University). Please alter if needed.

Please amend the manuscript submission data (via Edit Submission) to include author J Aluoch and R Kantor

This has been corrected in the manuscript submission data.

Please amend your authorship list in your manuscript file to include author Josephine Alouch

Done.

One of the noted authors is a group or consortium [for the RESPECT (RESistance in a PEdiatric CohorT) Study]. In addition to naming the author group, please list the individual authors and affiliations within this group in the acknowledgments section of your manuscript. Please also indicate clearly a lead author for this group along with a contact email address.

Upon review, we decided not to include the RESPECT Study group/consortium author as the authors within this study group are reflected in the individual authors names. We have now removed this from the manuscript submission data.

Please upload a copy of Figure 3a and 3b, to which you refer in your text on pages 19 and 22. If the figure is no longer to be included as part of the submission please remove all reference to it within the text.

We have corrected the Figure and Table references within the text. There are no Figures 3a or 3b.

If the reviewer comments include a recommendation to cite specific previously published works, please review and evaluate these publications to determine whether they are relevant and should be cited. There is no requirement to cite these works unless the editor has indicated otherwise.

Noted and done.

Additional Editor Comments:

TITLE: I would suggest the title to reflect the study population because it includes both children and adolescents (10-14 yrs). Although you have a well characterized cohort, I don't have the impression considering the follow up time that using the word "long-term" in the title is correct.

We have changed the title according to these suggestions. It now reads: “Adherence to antiretroviral therapy and drug resistance impact evolving viral suppression among a cohort of children and adolescents living with perinatal HIV infection in western Kenya”

ABSTRACT: As explained in comment 1 below, please state specific objective. As explained in the methods section below, clarify these issues of defining treatment failure as viral load (VL) >1,000copies/mL, ensure consistency with guidelines. For DR, indicate the gene and the sequencing methods. The design study should be clear, indicating study periods. Revise conclusion with respect to the clearly specified objectives.

The abstract has been edited per the requests, to include a clear objective, and revised methods and conclusions.

INTRODUCTION: The current objective is presented broadly and does not clearly reflect the analytical strategy described later in the manuscript. Please revise the Introduction to state a precise and focused primary objective (and secondary objectives if applicable) that focus on this stage of the study.

We have now restated the objective in both the Introduction and Abstract. In the Introduction, we have stated as follows: “Our objectives here were to comprehensively characterize ART adherence among CALWH receiving routine clinical care and to demonstrate its impact on treatment failure and HIV drug resistance.

I suggest you strengthen the rationale with: Why these historical samples (collected in 2010–2013) are relevant today and how the combination of MEMS, plasma drug levels, and treatment interruption measures adds value beyond existing evidence.

We appreciate this suggestion and have now elaborated on both the relevance of these historical samples and how the combined and detailed strategies for adherence evaluations (MEMS, plasma drug levels, and treatment interruption measures) add value. We have substantially rewritten the Introduction with these important comments in mind and believe that the rationale is strengthened as indicated here. Several portions of the Discussion also discuss the significance and value of the comprehensive adherence measurement strategies trialed here.

There is substantial repetition across “Study Setting,” “Study Design,” “CAMP study procedures,” and “Sample procedures.” Examples include: repeating the eligibility criteria, repeating VL/genotyping testing descriptions and others.

Provide complete details on sample storage and handling. Because all plasma samples were collected between 2010–2013 and tested years later under new funding, it is essential to document:

• storage temperature (−80°C???),

• storage duration

• freezer monitoring and quality assurance measures,

• whether any samples experienced freeze–thaw cycles

• how stored samples were managed prior to testing.

These details are needed to interpret the validity of VL, DRM, and drug-level assays.

We appreciate this careful review and have made edits accordingly. We have edited to remove the repetition across these settings. We have also now added the additional details requested on sample storage and handling at the end of the ‘Laboratory Methods’ section of the Methods, which now reads: ‘All samples were consistently stored at -80 °C in Kenya and the United States until testing, with minimal freeze-thaw cycles and continuous freezer monitoring and quality assurance. Samples were shipped between continents exclusively on dry ice and handled according to best laboratory practices at all sites. Prior to testing, samples were thawed once and tested immediately to minimize degradation, ensuring validity for viral load, drug resistance, and drug-level analyses.’

Provide Timeline for Laboratory Testing. The manuscript states that testing occurred after new funding but does not specify when. Please indicate the period during which VL, drug levels, and genotyping were performed. In addition, indicate the assay failure rate of each test, this will an idea about the sample integrity. Ensure transparency in reporting by stating for example whether storage conditions or RNA degradation played a role.

The new funding began in 2016 (now detailed in the manuscript). Viral load testing, was performed between 2016-2018, with an assay failure rate of 0%. Drug level testing was performed between 2019-2020, with an assay failure rate of 0%. Drug resistance testing was performed between 2018-2019, with an assay failure rate of 16% when VL>1,000 copies/mL. These details are now included in the manuscript in the Methods and Results sections.

The authors used several methods to measure adherence. While this might be useful, expand the Rationale for Multiple Adherence Measures.

We have now expanded the rationale for using multiple adherence measures in the section describing the Adherence Measures.

Streamline Redundant Sections between Study Setting, Study Design, and Original CAMP Procedures, and report especially relevant information directly to this study.

We have attempted to streamline and eliminate the redundancies throughout these sections, as you will see from the tracked changes.

9. Clarify the use of standard terminology throughout the paper. For viral load outcomes, ensure correct usage:

• “Suppressed” = <1000 copies/mL???

• “Undetectable” = <40 copies/mL???

TF is mistakenly taken for viral non suppression in some sentences. Please ensure consistency with known definition in the field (WHO or national guidelines).

As noted, we used World Health Organization definitions and defined detectable VL as > 40 copies/mL, and treatment failure (TF) as >1000 copies/mL. Thus, undetectable was <40 copies/mL and treatment success or suppression would, indeed, be <1000 copies/mL. We have corrected any instances where TF and viral non-suppression were described inappropriately in the Results.

10. Tables and figures: Overall, the tables and figures require clearer labelling, consistent variable naming, explicit definitions of numerical values and abbreviations, improved legends, and removal of ambiguous or unexplained formatting elements. These changes are essential to ensure that readers can correctly interpret the results and that the presentation aligns with the variables and methods described in the manuscript.

The tables and figure legends have been substantially revised as requested. Please also see responses to each specific comment below

Specifically:

- Improve clarity and completeness of row and variable labels

The formatting and labeling in all tables were substantially edited and improved. All of the numbers in the tables are identical to those in the submitted manuscript.

- Many tables contain missing, incomplete, or ambiguous row names (e.g., “value,” blank rows, or unclear variable titles).

These ambiguities have been removed and footnotes added to all tables. For example, the 1st two columns of the original tables were combined into one column with the variable in bold and the levels indented for clarification, and we omitted the unclear column labeled “value”. The footnotes also provide additional information about the variables and a list of all abbreviations used. For example, on Table 1 we added a footnote for the observed regimens in the “Other” category.

- All row labels should fully correspond to the variables defined in the Methods (e.g., MEMS %, number of >48h interruptions, BD1/BD2 VL, etc.).

We have done this both within the tables and also added additional details in the footnotes. This is particularly relevant for Table 4 (combined Table 4a and 4b) and the new supplemental Tables D, E and F.

The variable changes are as follows:

Changed “Questionnaire score” to “Adherence questionnaire score”

Changed “% MEMS adherence” to “% adherence using MEMS”

Changed “Number >48-hour breaks per week (MEMS)” to “Number treatment interruptions >48 hours standardized by number of weeks in period”

Changed “Max Tx interruption (log10, MEMS)” to “Duration (hours) of maximum treatment interruption”

Changed “No interruption >48 hrs” to “No treatment interruption >48 hrs”

- Clearly indicate what numerical values represent. Across several tables, it is unclear whether the numbers indicate counts (n), percentages, means (SD), or effect measures (OR/RR with CI).

We have done this both within the footnotes and alongside the variables themselves.- Column headers or footnotes must explicitly define each type of value.

The column headings and footnotes now include the summary measure or estimate provided in each cell of the tables.

- Standardize use of parentheses and statistical notation. Bracketed values appear without explanation in some tables (particularly Table 3). Every parenthetical value (SD, IQR, 95% CI, transformed variable) should be clearly identified.

We addressed this and are hopeful the new table formatting makes them much more clear.

- Ensure consistent and descriptive variable naming. Some variables appear truncated or unclear (“value???” or inconsistent acronyms). All variables should be fully spelled out and consistent with terminology used elsewhere in the manuscript.

We have made the variables consistent and better spelled out and fully elaborated on them in the footnotes.

- All tables should include a footnote defining abbreviations (e.g., BD1, BD2, TF, DRM, VL, MEMS).

We have done this.

- Effect estimates/confidence intervals, and p-values should appear in separate columns to improve readability.

Table 4 has been modified so that the p-values have separate columns.

- The row labeled “Log10 VL at BD1 (see Figure 3b)” should be renamed simply ““Log10 VL at BD1. ”Any cross-referencing should be placed in the caption, not inside the table body.

We have carefully revised the Tables and Figures, consistent with the helpful suggestions above.

Discussions:

11. Deepen Interpretation of Interruption Patterns (NNRTI pharmacokinetic profiles???, Selective drug pressure dynamics???, etc....)

We appreciate the suggestion to deepen this interpretation in the Discussion section and have now added the following text to the end of this paragraph to read: “These results suggest relatively rapid resistance development in regimens anchored by antiretrovirals with low resistance barriers, such as NVP or EFV, and in regimens comprising drugs with differing half lives, where abrupt cessation can lead to functional mono- or dual-therapy and promote resistance emergence. The lack of DR mutations with longer ART gaps likely reflects discontinuation of all ART during that period and potential reversion of the virus to wild-type.”

12. Explicitly Translate Findings to Today’s DTG Era, this is a critical revision to see the implications of your results in current ART era. In particular, please include a clear discussion on:

- NNRTI-Specific Findings (Patterns of EFV/NVP resistance, NNRTI-associated adherence thresholds????)

- Relevance to DTG-Based Regimens (in particular, how historical NRTI/NNRTI resistance affects modern DTG backbones)

- Relevance for Adolescents Transitioning From NNRTIs to DTG

This section is essential for framing the manuscript’s modern clinical value.

We have attempted to clarify and deepen the Discussion around each of these important points.

13. Extend the limitations with long-term sample storage, lack of contemporaneous laboratory testing, and missing data patterns and implications for bias.

We added these important limitations to the relevant Discussion paragraph, and the added sentence reads: “Finally, samples were stored for extended periods, precluding contemporaneous testing and potentially contributing to degradation and missing data that could affect interpretation.”.

CONCLUSIONS

14. Revise the Conclusion to Match Revised Objectives, after clarifying objectives (Highlight the key insights that remain relevant in today’s ART landscape).

The Conclusion has been revised:

In conclusion, comprehensively assessed poor adherence among Kenyan children and adolescents living with HIV was associated with longitudinal treatment failure and drug r

Attachment Submitted filename: Response to Reviewers.docx

# Peer review: Decision Letter 1

**DOI:** 10.1371/journal.pone.0355492.r003

**Authors:** YAGAI BOUBA

22 Jul 2026

Adherence to antiretroviral therapy and drug resistance impact evolving viral suppression among a cohort of children and adolescents living with perinatal HIV infection in western Kenya

PONE-D-25-21785R1

Dear Dr. Vreeman,

We’re pleased to inform you that your manuscript has been judged scientifically suitable for publication and will be formally accepted for publication once it meets all outstanding technical requirements.

Within one week, you’ll receive an e-mail detailing the required amendments. When these have been addressed, you’ll receive a formal acceptance letter and your manuscript will be scheduled for publication.

An invoice will be generated when your article is formally accepted. Please note, if your institution has a publishing partnership with PLOS and your article meets the relevant criteria, all or part of your publication costs will be covered. Please make sure your user information is up-to-date by logging into Editorial Manager at Editorial Manager® and clicking the ‘Update My Information' link at the top of the page. For questions related to billing, please contact billing support.

If your institution or institutions have a press office, please notify them about your upcoming paper to help maximize its impact. If they’ll be preparing press materials, please inform our press team as soon as possible -- no later than 48 hours after receiving the formal acceptance. Your manuscript will remain under strict press embargo until 2 pm Eastern Time on the date of publication. For more information, please contact onepress@plos.org.

Kind regards,

YAGAI BOUBA, PhD

Academic Editor

PLOS One

Additional Editor Comments (optional):

Dear Authors,

Thank you for your careful revision of the manuscript. The major concerns raised during the previous round of review have been satisfactorily addressed, and the manuscript has been substantially strengthened. In particular, the additional justification regarding the use of the older samples, the clarifications provided in the Methods section, and the discussion of the remaining limitations have improved the overall quality and transparency of the study.

Please consider addressing the following minor comments from the reviewer:

These are minor suggestions.

Reviewers' comments:

Reviewer's Responses to Questions

**Comments to the Author**

Reviewer #1: All comments have been addressed

**********

2. Is the manuscript technically sound, and do the data support the conclusions?

Reviewer #1: Yes

**********

3. Has the statistical analysis been performed appropriately and rigorously?

Reviewer #1: Yes

**********

4. Have the authors made all data underlying the findings in their manuscript fully available?

The PLOS Data policy

Reviewer #1: Yes

**********

5. Is the manuscript presented in an intelligible fashion and written in standard English?

Reviewer #1: Yes

**********

Reviewer #1: I am satisfied with the revised version of the manuscript. The article is well written, and the authors have adequately addressed most of the comments I raised in my previous review. I therefore have only minor comments, which are outlined below.

Overall Comments

Please ensure that all in-text citations are placed before punctuation marks, not after.

Please maintain consistent use of acronyms throughout the manuscript. For example, terms such as TF, DR, and DRMs are sometimes spelled out in full and other times used as acronyms, even within the same paragraph.

Abstract

Please define the acronym "MEMs" (Medication Event Monitoring Systems) at first mention.

In the results section of the abstract, the period appears as "between 2020-2013" – please correct this to "between 2010-2013".

Methods

I recommend moving the "Laboratory methods" section above the "Outcomes" section. Additionally, for clarity, I suggest describing each laboratory method (viral load testing, drug level testing, and genotypic resistance testing) in its own paragraph.

Results

I was not able to see Figure 1. It seems it has been included as a supplementary file (S1_Fig1) rather than as a main figure. Please correct this.

I could not locate Figure 2 in the manuscript as well – only the supplementary figure is visible, and it does not appear to contain Fig 2a or Fig 2b. I may have missed it, but I kindly ask the authors to double-check that all figures are correctly included.

References

References are duplicated.

**********

what does this mean?). If published, this will include your full peer review and any attached files.

If you choose “no”, your identity will remain anonymous but your review may still be made public.

**Do you want your identity to be public for this peer review?**  For information about this choice, including consent withdrawal, please see our Privacy Policy

Reviewer #1: No

**********

# Editor report: Acceptance letter

**DOI:** 10.1371/journal.pone.0355492.r004

**Authors:** YAGAI BOUBA

PONE-D-25-21785R1

PLOS One

Dear Dr. Vreeman,

I'm pleased to inform you that your manuscript has been deemed suitable for publication in PLOS One. Congratulations! Your manuscript is now being handed over to our production team.

At this stage, our production department will prepare your paper for publication. This includes ensuring the following:

* All references, tables, and figures are properly cited

* All relevant supporting information is included in the manuscript submission,

* There are no issues that prevent the paper from being properly typeset

You will receive further instructions from the production team, including instructions on how to review your proof when it is ready. Please keep in mind that we are working through a large volume of accepted articles, so please give us a few days to review your paper and let you know the next and final steps.

Lastly, if your institution or institutions have a press office, please let them know about your upcoming paper now to help maximize its impact. If they'll be preparing press materials, please inform our press team within the next 48 hours. Your manuscript will remain under strict press embargo until 2 pm Eastern Time on the date of publication. For more information, please contact onepress@plos.org.

You will receive an invoice from PLOS for your publication fee after your manuscript has reached the completed accept phase. If you receive an email requesting payment before acceptance or for any other service, this may be a phishing scheme. Learn how to identify phishing emails and protect your accounts at https://explore.plos.org/phishing.

If we can help with anything else, please email us at customercare@plos.org.

Thank you for submitting your work to PLOS One and supporting open access.

Kind regards,

PLOS One Editorial Office Staff

on behalf of

Dr. YAGAI BOUBA

Academic Editor

PLOS One
